# The Interplay between Pathophysiological Pathways in Early-Onset Severe Preeclampsia Unveiled by Metabolomics

**DOI:** 10.3390/life12010086

**Published:** 2022-01-07

**Authors:** Lina Youssef, Francesca Crovetto, Rui Vasco Simoes, Jezid Miranda, Cristina Paules, Miquel Blasco, Marta Palomo, Héctor García-Calderó, Olga Tura-Ceide, Ana Paula Dantas, Virginia Hernandez-Gea, Pol Herrero, Núria Canela, Josep Maria Campistol, Joan Carles Garcia-Pagan, Maribel Diaz-Ricart, Eduard Gratacos, Fatima Crispi

**Affiliations:** 1 BCNatal|Fetal Medicine Research Center, Hospital Clínic and Hospital Sant Joan de Déu, Institut d’Investigacions Biomèdiques August Pi i Sunyer (IDIBAPS), University of Barcelona, 08036 Barcelona, Spain; lyoussef@clinic.cat (L.Y.); fcrovetto@clinic.cat (F.C.); rui.simoes@research.fchampalimaud.org (R.V.S.); jezidmiranda@gmail.com (J.M.); cristinapaules@hotmail.com (C.P.); gratacos@clinic.cat (E.G.); 2 Champalimaud Research, Champalimaud Centre for the Unknown, 1400-038 Lisbon, Portugal; 3 Nephrology and Renal Transplantation Department, Hospital Clínic, Centro de Referencia en Enfermedad Glomerular Compleja del Sistema Nacional de Salud (CSUR), University of Barcelona, 08036 Barcelona, Spain; MIBLASCO@clinic.cat (M.B.); JMCAMPIS@clinic.cat (J.M.C.); 4 Josep Carreras Leukaemia Research Institute, Hospital Clinic, University of Barcelona, 08036 Barcelona, Spain; mpalomo@carrerasresearch.org; 5 Hematopathology, Centre Diagnòstic Biomèdic (CDB), Hemotherapy-Hemostasis Department, Hospital Clinic, Institut d’Investigacions Biomèdiques August Pi i Sunyer (IDIBAPS), University of Barcelona, 08036 Barcelona, Spain; mdiaz@clinic.cat; 6 Barcelona Endothelium Team (BET), 08036 Barcelona, Spain; 7 Barcelona Hepatic Hemodynamics Laboratory, Liver Unit, Hospital Clinic, Institut d’Investigacions Biomèdiques August Pi i Sunyer (IDIBAPS), University of Barcelona, 08036 Barcelona, Spain; HGARCIA@clinic.cat (H.G.-C.); VIHERNANDEZ@clinic.cat (V.H.-G.); JCGARCIA@clinic.cat (J.C.G.-P.); 8 Centro de Investigación Biomédica en Red de Enfermedades Hepáticas y Digestivas (CIBEREHD), Health Care Provider of the European Reference Network on Rare Liver Disorders (ERN-Liver), 08036 Barcelona, Spain; 9 Department of Pulmonary Medicine, Hospital Clínic, Institut d’Investigacions Biomèdiques August Pi i Sunyer (IDIBAPS), University of Barcelona, 08036 Barcelona, Spain; TURA@clinic.cat; 10 Biomedical Research Networking Center on Respiratory Diseases (CIBERES), Instituto de Salud Carlos III, 28029 Madrid, Spain; 11 Girona Biomedical Research Institute—IDIBGI, 17190 Girona, Spain; 12 Cardiovascular Institute, Hospital Clinic, Institut d’Investigacions Biomèdiques August Pi i Sunyer (IDIBAPS), University of Barcelona, 08036 Barcelona, Spain; ADANTAS@clinic.cat; 13 Eurecat, Centre Tecnològic de Catalunya, Centre for Omic Sciences (COS), Joint Unit Universitat Rovira i Virgili-EURECAT, Unique Scientific and Technical Infrastructures (ICTS), 43204 Reus, Spain; pol.herrero@agilent.com (P.H.); nuria.canela@eurecat.org (N.C.); 14 Centre for Biomedical Research on Rare Diseases (CIBER-ER), Instituto de Salud Carlos III, 28029 Madrid, Spain

**Keywords:** early-onset preeclampsia, fetal growth restriction, LC-MS/MS, maternal serum, preeclampsia, metabolomics, severe preeclampsia

## Abstract

Introduction: Preeclampsia is a multi-system disorder unique to pregnancy responsible for a great part of maternal and perinatal morbidity and mortality. The precise pathogenesis of this complex disorder is still unrevealed. Methods: We examined the pathophysiological pathways involved in early-onset preeclampsia, a specific subgroup representing its most severe presentation, using LC-MS/MS metabolomic analysis based on multi-level extraction of lipids and small metabolites from maternal blood samples, collected at the time of diagnosis from 14 preeclamptic and six matched healthy pregnancies. Statistical analysis comprised multivariate and univariate approaches with the application of over representation analysis to identify differential pathways. Results: A clear difference between preeclamptic and control pregnancies was observed in principal component analysis. Supervised multivariate analysis using orthogonal partial least square discriminant analysis provided a robust model with goodness of fit (R^2^X = 0.91, *p* = 0.002) and predictive ability (Q^2^Y = 0.72, *p* < 0.001). Finally, univariate analysis followed by 5% false discovery rate correction indicated 82 metabolites significantly altered, corresponding to six overrepresented pathways: (1) aminoacyl-tRNA biosynthesis; (2) arginine biosynthesis; (3) alanine, aspartate and glutamate metabolism; (4) D-glutamine and D-glutamate metabolism; (5) arginine and proline metabolism; and (6) histidine metabolism. Conclusion: Metabolomic analysis focusing specifically on the early-onset severe form of preeclampsia reveals the interplay between pathophysiological pathways involved in this form. Future studies are required to explore new therapeutic approaches targeting these altered metabolic pathways in early-onset preeclampsia.

## 1. Introduction

Preeclampsia is a multi-system disorder unique to pregnancy characterized by new-onset elevated blood pressure associated with proteinuria occurring after 20 weeks of gestation [1]. This complex disease affects 2–5% of pregnancies and is a major cause of maternal and perinatal morbidity and mortality [2,3]. However, the precise pathogenesis of preeclampsia is still unrevealed. Although abnormal placentation is known to play a central role especially in severe cases of preeclampsia [4,5], it is assumed that preeclampsia is a spectral disorder with different underlying etiologies, all converging into a seemingly identical clinical presentation that affects maternal health [6]. This heterogeneity strongly limits our understanding of the underlying etiology and the identification of therapeutic targets. The distinct forms of this disorder are recognized as early and late-onset preeclampsia before or after 34 weeks of gestation, respectively. Early-onset preeclampsia is less common than the late-onset form. However, it is more severe and frequently associated with maternal and perinatal complications [7]. Understanding the pathophysiology of early-onset severe preeclampsia is paramount for improving its clinical management, as well as detecting potential therapeutic targets for this subtype of preeclampsia.

Metabolomic-focused studies can contribute considerably to our understanding of complex multi-organ disorders by identifying biochemical mechanisms that are involved in their pathophysiology. Indeed, investigating and quantifying single metabolites offer a quite sensitive measure of the biochemical status of the studied subjects [8]. In addition, the interaction between these metabolites might uncover metabolic signatures of a disease or disease subtype [8]. Few studies have been conducted so far to examine the metabolomic profile of preeclampsia through maternal blood samples collected near delivery [9,10,11,12,13,14,15], but their results are difficult to interpret due to the high degree of sample heterogeneity and due to a focus on individual metabolites rather than examining the whole picture of activated pathways. In a previous study, we analyzed blood metabolomic profiles of mothers and fetuses at delivery using nuclear magnetic resonance approach and demonstrated that cases of preeclampsia with fetal growth restriction exhibit the most disturbed metabolomic profiles [16]. To further explore these complex alterations and pathophysiological pathways involved, we have now studied a well-defined subgroup of early-onset severe preeclampsia, typically associated with fetal growth restriction, using liquid chromatography tandem mass spectrometry (LC-MS/MS) metabolomics–a highly sensitive technique performed with multi-level extraction of lipids and small metabolites from maternal blood samples. Thus, our aim in this study was to identify the metabolomic fingerprint of early-onset severe preeclampsia and associated pathways underlying the pathogenesis of this disorder.

## 2. Results

### 2.1. Baseline and Perinatal Characteristics

Table 1 displays baseline characteristics and perinatal outcomes of the pregnancies included in this study. Baseline characteristics of pregnant women were comparable among the study groups. Ovum donation had not been used to achieve pregnancy in any of the three preeclamptic pregnancies achieved by assisted reproductive technologies. As expected, pregnancies complicated by preeclampsia presented altered feto-placental Doppler parameters. Liver enzymes including aspartate aminotransferase (AST), alanine aminotransferase (ALT), and gamma-glutamyl transferase (GGT) were higher in preeclampsia. One preeclamptic patient presented abnormal creatinine concentration, four patients presented abnormal AST and ALT concentrations and one patient presented high bilirubin and low platelets according to the cutoffs used clinically. In preeclampsia, average gestational age at the time of delivery was 32 weeks, up to 80% of deliveries were achieved through cesarean sections and all neonates were admitted to the neonatal intensive care unit, exhibiting subsequently one case of neonatal mortality. Two cases of preeclampsia had the diagnosis of hemolysis, elevated liver enzymes and low platelets (HELLP) syndrome.

### 2.2. Metabolomics Results

**Figure 1:**
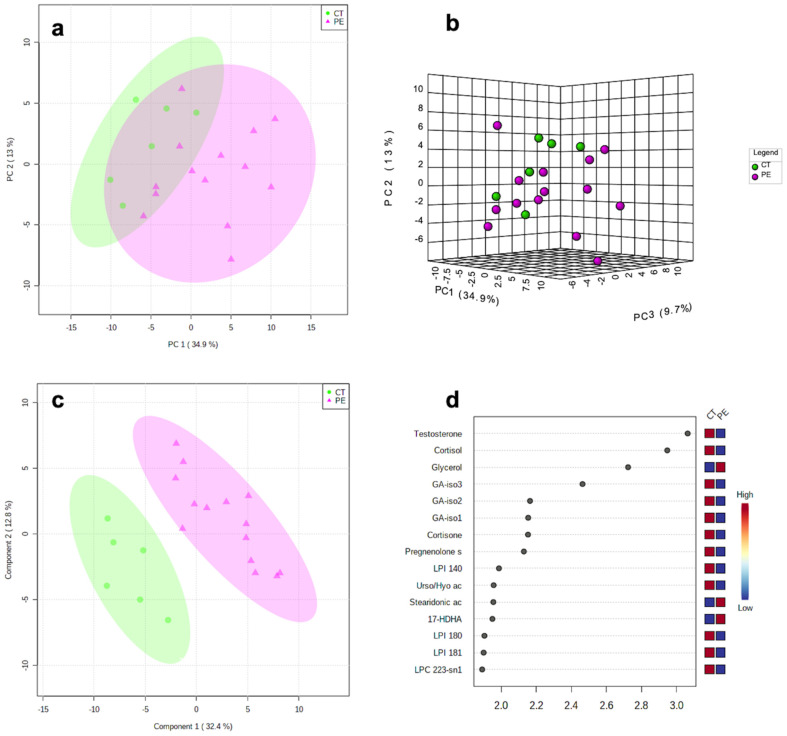
(**a**) 2-dimensional and (**b**) 3-dimensional principal component analysis (PCA) scores plots. (**c**) Partial least squares discriminant analysis (PLS-DA) scores plot between components 1 and 2. The explained variance is shown in brackets. Controls (CT) are presented in green circles and preeclampsia cases (PE) in violet triangles. (**d**) Top 15 most important metabolites that contribute to group separation identified through PLS-DA ranked by variable importance in projection (VIP) scores. The right heatmap shows the mean intensity variable in the respective group, with red and blue indicating high and low metabolite levels, respectively. GA, glyco-cholic acid; HDHA, hydroxy-docosahexaenoic acid; LPC, lyso-phosphatidylcholines; LPE, Lyso-phosphatidylethanolamine; LPI, lyso-phosphatidylinositol; NA, non-applicable; Pregnenolone s, pregnenolone sulfate; Stearidonic ac, stearidonic acid; Urso/Hyo ac, urso-deoxycholic/hyo-deoxycholic acid.

383 metabolites (out of 400) were present in ≥70% of the samples (complete results dataset is provided in Supplementary Materials Table S1). Figure 1a,b shows a clear separation between preeclampsia and controls through the principal component analysis (PCA). The first and second components explained 34.9% and 13% of the variance between cases and controls, respectively. Figure 1c demonstrated the separation between the study groups by the partial least squares discriminant analysis (PLS-DA). Figure 1d demonstrates the top 15 most important metabolites responsible for class separation. The contribution of each metabolite is demonstrated by the distance from the Y-axis (the greater the distance, the greater the contribution). The heatmap on the right side of this plot also indicates if the concentration of this metabolite is higher or lower in preeclampsia compared to controls. The model obtained by orthogonal projection to latent structures discriminant analysis (OPLS-DA) showed a high goodness of fit (R2X = 0.915, *p* = 0.003) and a strong predictive ability (Q^2^Y = 0.718, *p* = 0.001, 1 predictive + 1 orthogonal components), i.e., the model explains more than 90% of the variation between the study groups with a predictive ability of 72%). The two cases of HELLP syndrome were not separated from other severe preeclamptic pregnancies.

**Figure 2:**
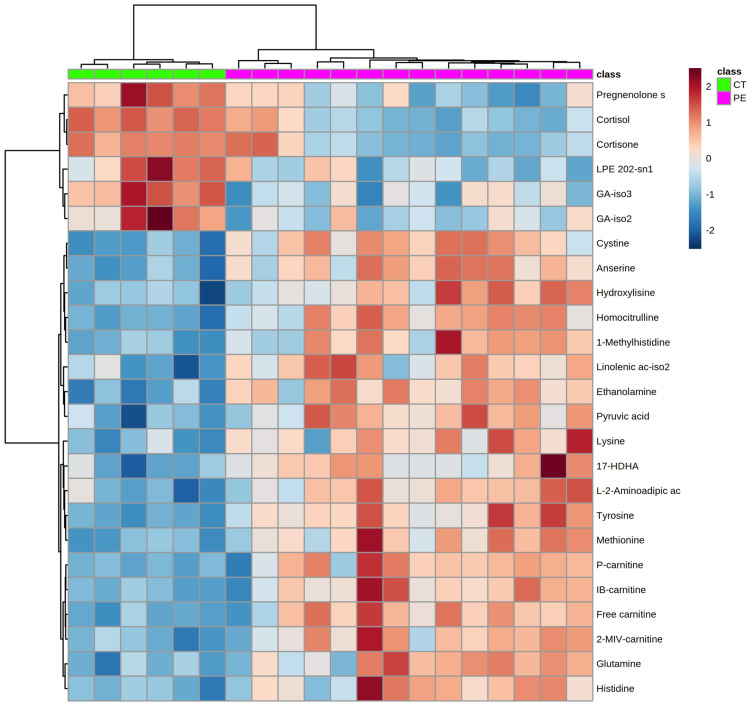
Unsupervised hierarchical clustering considering the top 25 metabolites selected from univariate analysis. A z-score transformation was performed on the intensity of each metabolite across all samples and the heatmap displays each sample z-score. Rows represent metabolites and columns represent samples. Both metabolites and samples are clustered by Euclidean distance and Ward linkage. 1-Methylhistidine (HMDB00001); 2-MIV-carnitine, 2-methyl-butyryl-carnitine/isovaleryl-carnitine/valeryl-carnitine (HMDB00688); Aminoadipic ac, aminoadipic acid (HMDB00510); Anserine (HMDB00194); Cortisol (HMDB00063); Cortisone (HMDB02802); Cystine (HMDB00192); Ethanolamine (HMDB00149); Free carnitine (HMDB00062); GA, glyco-cholic acid (HMDB0000138); Glutamine (HMDB00641); HDHA, hydroxy-docosahexaenoic acid (NA); Histidine (HMDB00177); IB-carnitine, iso-butyryl-carnitine/butyryl-carnitine (HMDB02013); Homo-citrulline (HMDB00679); Hydroxylysine (HMDB00450); L-2-Aminoadipic ac, L-2-Aminoadipic acid (HMDB00510); Linolenic ac, linolenic acid (HMDB00673); LPE, lyso-phosphatidylethanolamine (HMDB11483); Lysine (HMDB00182); Methionine (HMDB00696); Pregnenolone s, pregnenolone sulfate (HMDB00774); P-carnitine, propionyl-carnitine (HMDB00824); Pyruvic acid (HMDB00243); Tyrosine (HMDB00158).

Univariate analysis revealed 82 statistically different metabolites in preeclampsia vs. controls (Table 2), in agreement with the multivariate analysis. Hierarchical clustering analysis (HCA) considering the top 25 metabolites showed 2 clusters in correspondence with the study groups (Figure 2).

**Figure 3:**
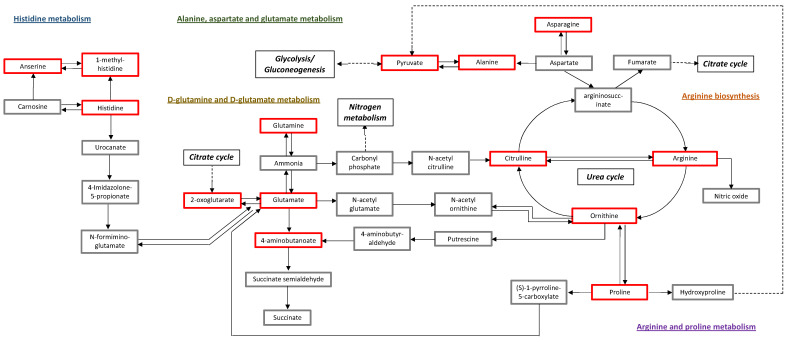
Proposed metabolome view with significant pathways according to the pathway enrichment analysis. The meaning of compound colors within the pathways is as follows: grey means those metabolites are not detected as significantly different in our data and are used as background for enrichment analysis; red means that the metabolites are in the data with significantly higher levels in early-onset severe preeclampsia compared to controls. KEGG database (http://www.kegg.jp/kegg/kegg1.html, accessed on 11 August 2021) was the source of the proposed metabolic pathways.

The over representation analysis based on the 82 differentially expressed metabolites resulted in the significant over representation of eight pathways out of 40 pathways identified: (1) aminoacyl-tRNA biosynthesis; (2) arginine biosynthesis; (3) alanine, aspartate and glutamate metabolism; (4) valine, leucine and isoleucine biosynthesis; (5) linoleic acid metabolism; (6) D-glutamine and D-glutamate metabolism; (7) arginine and proline metabolism; and (8) histidine metabolism (Table 3 and Figure 3). However, pathways 4 and 5 (valine, leucine and isoleucine biosynthesis and linoleic acid metabolism) correspond to essential amino and fatty acids, and therefore are not functional in humans. Thus, pathways 4 and 5 were further excluded from the results leaving six differential pathways in early-onset severe preeclampsia compared to controls. Lipid data showed higher triglycerides and reduced glyco-cholic acid, lyso-phosphatidylethanolamines, lyso-phosphatidylcholines, lyso-phosphatidylinositols, pregnenolone sulfate, dehydroepiandrosterone sulfate, testosterone, cortisol and cortisone. The cortisol:cortisone ratio was significantly lower in preeclampsia (2.63 ± 0.7 vs. 7.09 ± 1.39 in controls, *p* < 0.001).

## 3. Discussion

This study displays the significant changes in the metabolome of pregnant women with early-onset severe preeclampsia, showing for the first time a comprehensive set of circulating metabolites and associated pathophysiological pathways. The most discriminative pathways were related to the metabolism of specific amino acids such as arginine, alanine, aspartate, glutamate, proline and histidine, in addition to an altered lipid profile.

Among the main pathways identified in the current study are the arginine biosynthesis and metabolism. Indeed, arginine is an essential molecule in the pathophysiology of preeclampsia since it is the precursor of nitric oxide, a potent endothelial-derived vasodilator [17]. A reduced activity of the nitric oxide might contribute to the clinical features of preeclampsia such as vasoconstriction and endothelial dysfunction [17]. In the literature, controversial results have been reported for arginine levels in preeclampsia, likely to be due to differences in the studied population and the methodology used [18,19]. Few trials have reported a potential role for arginine in the prevention and treatment of preeclampsia [20]. Our data show higher levels of arginine in early-onset severe preeclampsia and further demonstrate its interaction with other altered and non-altered metabolites that might be targeted to stop the vicious cycle of activated pathophysiological pathways (Figure 3).

In the present study, alanine, aspartate, glutamate and glutamine metabolism were also identified among the principal metabolic pathways in early-onset severe preeclampsia. Essentially, the glutamine-cycling pathway plays a crucial role in the development of metabolic risk [21]. Abnormal glutamate metabolism suggests liver involvement in the global metabolic modulation since glutamate metabolism is linked to aminotransferase reactions [22]. Indeed, the metabolism of almost all amino acids is initiated by aminotransferases, and glutamate is produced by the transfer of the amino group [23]. AST and ALT are two aminotransferases widely used in monitoring preeclamptic patients since they become elevated in severe cases such as the most extreme phenotype of preeclampsia, the HELLP syndrome. Furthermore, it is known that women exposed to preeclampsia during pregnancy have an increased risk of metabolic syndrome later in life [24].

In addition, our data suggest that histidine metabolism is also disturbed in early-onset severe preeclampsia with high levels of histidine, 1-methylhistidine and other metabolites in line with the literature [12,18]. However, no detailed profiling of this pathway has been done previously in preeclampsia. Histidine is the precursor of carnosine which functions as an antioxidant and scavenger of reactive oxygen species and unsaturated aldehydes of cell membrane fatty acids formed due to peroxidation during oxidative stress [25]. Therefore, high levels of histidine might reflect exacerbated oxidative stress in early-onset severe preeclampsia [1,26].

Moreover, patients with early-onset severe preeclampsia exhibited higher concentrations of valine, leucine and isoleucine in agreement with our previous findings in a population of preeclamptic mothers with growth-restricted fetuses [16]. These three metabolites are essential amino acids that play a crucial role in energy metabolism. A combined profile of isoleucine, leucine, valine, tyrosine and phenylalanine is a good predictor of future diabetes [27]. These results might explain the link between preeclampsia and the two-fold increased risk of diabetes throughout life [28].

Furthermore, this study demonstrates the involvement of the essential fatty acid linoleic acid in early-onset severe preeclampsia. It is known that endothelial dysfunction during preeclampsia might be enhanced by the imbalance of vasoactive prostaglandins (prostacyclin:thromboxane ratio) causing vasoconstriction of small arteries and platelets activation [29]. Linoleic acid is the main precursor of arachidonic acid, the origin compound of prostaglandins and prostanoids. Prior trials investigated whether daily use of linoleic acid and calcium supplementation during the third trimester effectively prevents preeclampsia in high-risk women. No evidence was found that this supplementation influences the incidence of preeclampsia, the length-of-gestation, or the need for preterm delivery [30]. However, it has been demonstrated that daily treatment with aspirin, inhibits prostaglandin synthesis from the arachidonic acid, in high-risk women decreases the incidence of preterm preeclampsia [31].

On the other hand, lipid data seem to be highly disturbed in early-onset severe preeclampsia. It is well established that preeclampsia is a disease characterized by inflammation, complement activation, altered lipid metabolism and related oxidative stress [16,32,33,34]. Lipid classes are linked tightly during their synthesis, which may explain the similarity of observed alterations in most of them. Low phospholipid levels play a role in promoting cell membrane damage and inflammation. In preeclampsia, the decrease in pregnenolone sulfate, dehydroepiandrosterone sulfate and testosterone might be related to impaired placental steroidogenesis [35]. Moreover, low cortisol and cortisone are most likely to be due to enhanced glucocorticoid metabolism, in addition to intensified conversion of cortisol to its inactive form cortisone since the cortisol:cortisone ratio was also significantly lower in preeclampsia compared to controls [36]. This imbalance might contribute to high blood pressure in preeclampsia, obesity, renal disease and other hypertensive disorders [37]. Thus, based on previously published data we can speculate that disturbed lipid metabolism might be involved in the development of preeclampsia while altered pathways of amino acid metabolism are mainly involved in the clinical manifestation of this disease.

The prospective design of the current study and the recruitment of a well-characterized homogenous group of early-onset severe preeclampsia associated with fetal growth restriction with no chronic disorders are the principal strengths of this study. Moreover, the collection of maternal blood samples was achieved directly upon confirming the diagnosis in the cases and at matched gestational age in the controls. Fasting status was assured in all the participating patients. The processing, storage and analysis of the samples followed rigorous protocols. Among the limitations, we declare the relatively small number of cases and controls in this study and the importance of validating our results in larger cohorts and exploring the detected pathways in other phenotypes of preeclampsia such as late-onset preeclampsia and preeclampsia with no associated fetal growth restriction.

## 4. Materials and Methods

### 4.1. Study Population

This was a nested case-control study within the project “Targeting endothelial dysfunction in highly prevalent diseases–PIE15/00027”. Singleton pregnancies with a diagnosis of early-onset severe preeclampsia associated with fetal growth restriction who attended the Departments of Maternal-Fetal Medicine at BCNatal (Barcelona, Spain) between July 2016 and December 2017 were recruited prospectively. Preeclampsia was defined as high blood pressure (systolic blood pressure ≥ 140 mmHg and/or diastolic blood pressure ≥ 90 mmHg on two occasions, at least four hours apart) with proteinuria (≥300 mg/24 h or protein/creatinine ratio ≥ 0.3) developed after 20 weeks of gestation [38,39]. Early-onset preeclampsia was defined as disease onset before 34 weeks of gestation [7]. To include a homogenous group of complicated pregnancies, we recruited early-onset cases that needed elective delivery before 34 weeks of gestation indicated for severe preeclampsia which was considered upon presenting one or more of the following severity criteria [39]: blood pressure ≥160 mmHg systolic or ≥110 mmHg diastolic on two occasions at least 4 h apart, thrombocytopenia (<100,000/mm^3^), impaired liver function (elevated blood concentrations of liver enzymes to twice normal concentration and/or severe persistent right upper quadrant or epigastric pain unresponsive to medication and not accounted for by alternative diagnoses), progressive renal insufficiency (serum creatinine concentration > 1.1 mg/dL), pulmonary edema, new-onset cerebral or visual disturbances. HELLP syndrome was defined as preeclampsia associated with lactate dehydrogenase elevated to 600 u/L or more, AST and ALT elevated more than twice the upper limit of normal, and platelets count < 100,000/mm^3^ [39]. Pregnancy termination was by labor induction or cesarean section upon obstetric indication. Fetal growth restriction was defined as estimated fetal weight and birthweight below the 10th centile associated with either abnormal cerebroplacental ratio (<5th centile) or abnormal uterine arteries mean pulsatility index (>95th centile), or birthweight below the 3rd centile. Uncomplicated pregnancies with normotensive mothers and an appropriate for gestational age fetus -defined as estimated fetal weight and birthweight above the 10th centile- were randomly selected from our general population to be included as controls and matched with cases by maternal age, ethnicity, pre-gestational body mass index and gestational age at maternal blood draw (±2 weeks). Estimated fetal weight and birthweight centiles were calculated according to local standards [40]. In all pregnancies, gestational age was calculated based on the crown-rump length at first trimester ultrasound [41]. Pregnancies with chromosomal/structural anomalies or intrauterine infection were excluded. The history of pregestational diabetes, autoimmune, renal or coagulation disorders were also considered excluding criteria.

### 4.2. Data Collection

Maternal age, ethnicity, pregestational body mass index, chronic hypertension, parity, obstetric history, mode of conception and smoking status were collected at enrolment. 

Estimated fetal weight and feto-placental Doppler assessment was achieved in all the study participants. Ultrasound studies were performed using a Siemens Sonoline Antares (Siemens Medical Systems, Malvern, PA, USA) or a Voluson 730 Expert (GE Medical Systems, Milwaukee, WI, USA) with 6–4-MHz linear curved-array probes. Estimated fetal weight was calculated using the Hadlock formula [42] and centile based on local reference curves [40]. Fetoplacental Doppler examination followed standardized guidelines [43] included the uterine arteries [44], the umbilical artery [45], the fetal middle cerebral artery [45] and the ductus venosus [46] with the calculation of the cerebroplacental ratio [47]. Maternal biochemical profile was also assessed at the time of maternal blood draw, including the evaluation of renal (creatinine, urea, sodium, potassium) and liver (AST, ALT, GGT) function, uric acid, glucose, triglycerides, total cholesterol, fibrinogen and platelets count.

At the time of delivery, gestational age, birthweight, Apgar scores, umbilical artery pH, neonatal intensive care unit admissions and perinatal mortality were recorded.

### 4.3. Maternal Blood Sampling

Peripheral maternal blood was obtained by venipuncture within 24–48 h of preeclampsia diagnosis and at matched gestational age for controls. Sampling was done in the morning after fasting for 6–8 h at least. The samples were incubated for 30 min at room temperature to allow clotting and subsequently centrifuged at 1500 g for 10 min at 4 °C to separate the serum from clots. Afterwards, serum samples were transferred to acetonitrile treated tubes and immediately stored at −80 °C until assayed.

### 4.4. Metabolomic Analysis

The metabolomic analysis comprised the determination of lipids (lipidomics), amino acids and polar metabolites, achieved through a rigorous and well-established four-level extraction protocol. Lipidomic analysis was achieved by two methods, with methanol extraction and with choloform:methanol extraction (Folch method). Detailed methodology is provided in the Appendix A. This analysis was achieved by investigators blinded to the study group of each sample.

### 4.5. Statistical Analysis

Clinical data were analyzed with the statistical software STATA 14.2 (StataCorp LLC, College Station, TX, USA). Categorical data are displayed as percentages and continuous data as mean ± standard deviation or median (interquartile range) according to their distribution (normality was assessed using the Kolmogorov-Smirnov test). Statistical analysis for continuous variables included the use of student *t*-tests for normally distributed data or Mann Whitney U tests in non-normally distributed data. Fisher exact test was used for categorical variables. All reported *p*-values are two-sided. *p* < 0.05 was set for statistical significance.

For metabolomics data, statistical approach was performed using Metaboanalyst 4.0 (http://www.metaboanalyst.ca/, accessed on 11 August 2021). Initially, a multivariate modeling was applied including the use of unsupervised methods such as PCA, and supervised methods such as PLS-DA and OPLS-DA. A variable importance in projection (VIP) plot, which is a visual representation of the importance of the metabolites in discriminating the groups of interest, is provided. Secondly, for each protein a univariate Student’s *t*-test was performed and the Benjamini-Hochberg method was used to adjust *p* values for multiple testing with consideration of a 5% false discovery rate. An additional unsupervised HCA was performed based on the univariate results. Last, differential pathways were identified from an over representation analysis performed in Metaboanalyst using the Pathway Analysis option. This analysis was performed using a Fisher’s exact test to calculate the probability of finding at least a particular number of metabolites containing a biological term of interest in the given compound list based on the Kyoto Encyclopedia of Genes and Genome (KEGG) [48]. Detailed statistical approach is provided in the Appendix A.

## 5. Conclusions

In conclusion, we identified a complex mix of pathophysiological pathways in early-onset severe preeclampsia in this study. The main changes were attributed to amino acids, specifically arginine, alanine, aspartate, glutamate, valine, leucine, isoleucine, proline and histidine, and linoleic acid metabolism. The metabolomic profile observed in early-onset severe preeclampsia suggests the involvement of multiple pathways in its clinical manifestations, such as vasoconstriction, endothelial dysfunction, oxidative stress, complement activation, inflammation and predisposition to metabolic syndrome and diabetes. The interplay between the detected pathways may provide a better understanding of the underlying etiology for this specific phenotype of preeclampsia and unveil its impending therapeutic targets. Further research is warranted to develop effective therapeutics and investigate their usefulness in reducing maternal and perinatal complications in early-onset severe preeclampsia.

## Figures and Tables

**Table 1:** Baseline characteristics of the study population.

	Controls *n* = 6	Preeclampsia *n* = 14	*p*-Value
Maternal characteristics			
Age (years)	36.5 ± 2.9	34.1 ± 3.8	0.28 *
Caucasian ethnicity	5 (83.3)	6 (42.9)	0.10 ^ƒ^
Pre-gestational BMI (kg/m^2^)	22.2 ± 2.7	24.1 ± 4.1	0.38 *
Nulliparity	5 (83.3)	9 (64.3)	0.39 ^ƒ^
Previous preeclampsia	0 (0)	1 (7.1)	0.50 ^ƒ^
Chronic hypertension	0 (0)	1 (7.1)	0.50 ^ƒ^
Assisted reproductive technologies	0 (0)	3 (21.4)	0.22 ^ƒ^
Smoking during pregnancy	1 (16.7)	1 (7.1)	0.52 ^ƒ^
Fetal ultrasound assessment			
Gestational age at ultrasound (weeks)	26.6 ± 3.7	30.5 ± 2.6	0.013 *
Estimated fetal weight (g)	994 ± 496	1187 ± 328	0.30 *
Estimated fetal weight centile	34 (29–66)	1 (0–3)	<0.001 ^ω^
Uterine arteries mean PI (z score)	−0.53 ± 1.77	2.60 ± 2.08	0.002 *
Umbilical artery PI (z score)	−0.40 ± 0.32	1.27 ± 1.71	0.07 *
Middle cerebral artery PI (z score)	0.13 ± 1.09	−1.16 ± 0.99	0.027 *
Cerebroplacental ratio (z score)	−0.06 ± 0.89	−1.93 ± 1.6	0.027 *
Ductus venosus PI (z score)	−1 ± 0.79	−0.34 ± 1.29	0.44 *
Maternal biophysical and biochemical profile at the time of sampling			
Gestational age at blood draw (weeks)	30 ± 1.8	31.3 ± 1.8	0.16 *
Systolic blood pressure (mmHg)	150.5 ± 18.1	107.7 ± 8.3	<0.001 *
Diastolic blood pressure (mmHg)	89.3 ± 8	70.7 ± 3.3	<0.001 *
Creatinine (mg/dL)	0.42 ± 0.07	0.69 ± 0.17	0.001 *
Urea (mg/dL)	15.4 ± 4.2	40.4 ± 16.7	0.002 *
Glomerular filtration rate (mL/min)	85 ± 12.2	62.1 ± 8	<0.001 *
Sodium (mEq/L)	138.3 ± 1.2	136.4 ± 2.1	0.048 *
Potassium (mEq/L)	4.13 ± 0.21	4.61 ± 0.23	0.001 *
AST (u/L)	17 (15–23)	25 (16–48)	0.16 ^ω^
ALT (u/L)	15 (9–21)	27 (14–65)	0.07 ^ω^
GGT (u/L)	10 (7–11)	19 (15–28)	0.013 ^ω^
Uric acid (mg/dL)	3.33 ± 0.43	6.09 ± 1.25	<0.001 *
Glucose (mg/dL)	75.5 ± 7.1	87.8 ± 19.7	0.13 *
Triglycerides (mg/dL)	178.8 ± 67.7	260.6 ± 97.8	0.06 *
Total cholesterol (mg/dL)	277.2 ± 50.1	241.9 ± 54	0.16 *
Fibrinogen (g/L)	4.57 ± 0.98	3.44 ± 1.05	0.07 *
Platelets (10^3^/L)	271.8 ± 70.6	209.1 ± 71.9	0.09 *
Proteinuria (g/24 h)	-	2.6 (0.6–5.8)	-
Protein/creatinine ratio	-	2.2 (0.4–4.2)	-
Perinatal outcomes			
Gestational age at delivery (weeks)	40.3 ± 1.1	32 ± 1.9	<0.001 *
Cesarean section	1 (16.7)	11 (78.6)	0.018 ^ƒ^
Female sex	4 (66.7)	8 (57.1)	0.69 ^ƒ^
Birthweight (g)	3531 ± 410	1287 ± 267	<0.001 *
Birthweight centile	58 (41–80)	0 (0–1)	<0.001 ^ω^
APGAR score 5 min < 7	0 (0)	1 (7.1)	0.50 ^ƒ^
Umbilical artery pH	7.20 ± 0.07	7.20 ± 0.09	0.97 *

Data are presented as mean ± standard deviation, median (interquartile range) or *n* (%) as appropriate. BMI, body mass index; PI, pulsatility index; AST, aspartate aminotransferase; ALT, alanine aminotransferase; GGT, gamma-glutamyl transferase. *p* value was calculated by student *t* (*), Mann Whitney U (^ω^) or Fisher exact (^ƒ^) tests as appropriate.

**Table 2:** The 82 metabolites significantly different in preeclampsia vs. controls by univariate analysis considering false discovery rate correction (*q*-value < 0.05).

HMDB	Metabolite Name	Controls Mean (SD)	Preeclampsia Mean (SD)	*p*-Value	*q*-Value	Preeclampsia/Controls
HMDB00192	Cystine	−0.553 (0.167)	0.237 (0.251)	<0.0001	< 0.0001	Up
HMDB00149	Ethanolamine	−0.397 (0.160)	0.170 (0.174)	<0.0001	< 0.0001	Up
HMDB00158	Tyrosine	−0.431 (0.070)	0.185 (0.238)	<0.0001	< 0.0001	Up
HMDB00062	Free carnitine	−0.572 (0.138)	0.245 (0.393)	<0.0001	< 0.0001	Up
HMDB00194	Anserine	−0.361 (0.130)	0.155 (0.183)	<0.0001	0.0006	Up
HMDB00824	Propionyl-carnitine	−0.581 (0.079)	0.249 (0.464)	<0.0001	0.0006	Up
HMDB02013	Iso-butyryl-carnitine/ Butyryl-carnitine	−0.565 (0.120)	0.242 (0.465)	<0.0001	0.0011	Up
HMDB0000138	Glyco-cholic acid-iso3	0.819 (0.401)	−0.351 (0.450)	<0.0001	0.0014	Down
HMDB00679	Homo-citrulline	−0.614 (0.158)	0.263 (0.318)	<0.0001	0.0021	Up
HMDB00243	Pyruvic acid	−0.505 (0.282)	0.216 (0.300)	<0.0001	0.0034	Up
HMDB00774	Pregnenolone sulfate	0.708 (0.395)	−0.303 (0.426)	0.0001	0.0035	Down
HMDB00696	Methionine	−0.512 (0.144)	0.219 (0.351)	0.0001	0.0038	Up
HMDB00510	L-2-Aminoadipic acid	−0.561 (0.334)	0.240 (0.339)	0.0001	0.0038	Up
HMDB00673	Linolenic acid-iso2	−0.567 (0.368)	0.243 (0.341)	0.0002	0.0044	Up
HMDB00450	Hydroxylysine	−0.490 (0.292)	0.210 (0.354)	0.0002	0.0054	Up
HMDB00688	2-methyl-butyryl-carnitine/Isovaleryl-carnitine/Valeryl-carnitine	−0.562 (0.219)	0.241 (0.403)	0.0002	0.0060	Up
HMDB0000138	Glyco-cholic acid-iso2	0.719 (0.650)	−0.308 (0.374)	0.0003	0.0061	Down
HMDB00001	1-Methylhistidine	−0.524 (0.168)	0.224 (0.390)	0.0003	0.0064	Up
HMDB00182	Lysine	−0.374 (0.181)	0.160 (0.271)	0.0004	0.0069	Up
HMDB06547	Stearidonic acid	−0.650 (0.508)	0.278 (0.534)	0.0004	0.0069	Up
HMDB00904	Citrulline	−0.300 (0.080)	0.129 (0.341)	0.0004	0.0075	Up
HMDB00641	Glutamine	−0.396 (0.169)	0.170 (0.301)	0.0005	0.0075	Up
HMDB11483	LPE 20:2-sn1	0.616 (0.513)	−0.264 (0.380)	0.0005	0.0075	Down
HMDB00063	Cortisol	0.979 (0.168)	−0.420 (0.561)	0.0006	0.0089	Down
HMDB00168	Asparagine	−0.311 (0.127)	0.133 (0.352)	0.0006	0.0089	Up
HMDB00177	Histidine	−0.372 (0.132)	0.160 (0.299)	0.0006	0.0089	Up
HMDB00234	Testosterone	1.018 (0.787)	−0.436 (0.695)	0.0006	0.0089	Down
HMDB0000138	Glyco-cholic acid-iso1	0.716 (0.543)	−0.307 (0.500)	0.0007	0.0094	Down
HMDB00687	Leucine	−0.376 (0.195)	0.161 (0.294)	0.0007	0.0096	Up
HMDB00008	2-Hydroxybutyric acid	−0.611 (0.395)	0.262 (0.460)	0.0008	0.0098	Up
NA	LPI 18:1	0.631 (0.252)	−0.271 (0.740)	0.0008	0.0098	Down
HMDB00159	Phenylalanine	−0.366 (0.170)	0.157 (0.298)	0.0009	0.0104	Up
HMDB10383	LPC 16:1 e	−0.294 (0.166)	0.126 (0.235)	0.0009	0.0108	Up
HMDB00208	alpha-Ketoglutaric acid	−0.368 (0.208)	0.158 (0.306)	0.0013	0.0139	Up
NA	11.13-Eicosadienoic acid	−0.530 (0.377)	0.227 (0.418)	0.0013	0.0139	Up
HMDB00517	Arginine	−0.297 (0.132)	0.127 (0.367)	0.0013	0.0139	Up
NA	17-HDHA	−0.648 (0.384)	0.278 (0.443)	0.0013	0.0139	Up
HMDB00167	Threonine	−0.415 (0.295)	0.178 (0.353)	0.0015	0.0156	Up
HMDB00883	Valine	−0.344 (0.265)	0.147 (0.274)	0.0016	0.0157	Up
HMDB06528	w3-docosapentaenoic acid	−0.524 (0.416)	0.225 (0.435)	0.0022	0.0203	Up
HMDB00172	Isoleucine	−0.309 (0.123)	0.133 (0.289)	0.0022	0.0203	Up
HMDB00148	Glutamic acid	−0.262 (0.267)	0.112 (0.191)	0.0022	0.0203	Up
HMDB00214	Ornithine	−0.439 (0.261)	0.188 (0.394)	0.0023	0.0203	Up
HMDB00946	Urso-deoxycholic/ Hyo-deoxycholic acid	0.650 (0.394)	−0.279 (0.584)	0.0024	0.0204	Down
HMDB07132 HMDB07105 HMDB07051 HMDB07023 HMDB07024	DG 34:3	−0.411 (0.335)	0.176 (0.344)	0.0024	0.0204	Up
HMDB00190	Lactic acid	−0.295 (0.151)	0.126 (0.273)	0.0024	0.0204	Up
HMDB00826	Pentadecanoic acid	−0.440 (0.236)	0.189 (0.407)	0.0025	0.0206	Up
HMDB05474	TG 54:6	−0.517 (0.475)	0.221 (0.415)	0.0026	0.0206	Up
HMDB02231	Eico-senoic acid	−0.521 (0.332)	0.223 (0.474)	0.0027	0.0213	Up
HMDB00271	Sarcosine	−0.481 (0.338)	0.206 (0.436)	0.0030	0.0230	Up
HMDB00161	Alanine	−0.378 (0.185)	0.162 (0.367)	0.0033	0.0243	Up
HMDB12328	Trans-palmitoleic acid	−0.497 (0.244)	0.213 (0.607)	0.0033	0.0243	Up
HMDB06734	ChoE 202	0.320 (0.336)	−0.137 (0.252)	0.0034	0.0243	Down
NA	LPC 22:3-sn1	0.629 (0.343)	−0.269 (0.607)	0.0034	0.0243	Down
HMDB05447 HMDB10490 HMDB10502 HMDB10475 HMDB10489	TG 54:7	−0.543 (0.565)	0.233 (0.433)	0.0035	0.0243	Up
HMDB07972	PC 34:1 e	0.350 (0.286)	−0.150 (0.315)	0.0037	0.0249	Down
NA	LPI 18:2	0.624 (0.343)	−0.267 (0.609)	0.0037	0.0249	Down
HMDB04702	12.13-EpOME(9)	−0.461 (0.293)	0.198 (0.443)	0.0039	0.0254	Up
HMDB10392	LPC 20:2-sn2	0.516 (0.470)	−0.221 (0.455)	0.0041	0.0264	Down
HMDB0240261	LPI 18:0	0.633 (0.280)	−0.271 (0.643)	0.0042	0.0267	Down
HMDB31654	3-Aminobutanoic acid	−0.415 (0.227)	0.178 (0.414)	0.0042	0.0267	Up
HMDB10221	HODE-iso1	−0.512 (0.440)	0.219 (0.475)	0.0048	0.0295	Up
NA	LPI 22:4	0.548 (0.358)	−0.235 (0.546)	0.0049	0.0300	Down
NA	LPI 20:4	0.590 (0.305)	−0.253 (0.613)	0.0053	0.0320	Down
HMDB10386	LPC 18:2-sn2	0.453 (0.348)	−0.194 (0.446)	0.0056	0.0330	Down
HMDB00694	2-hydroxyglutaric acid	−0.346 (0.099)	0.148 (0.557)	0.0060	0.0349	Up
HMDB08287 HMDB08257 HMDB08318 HMDB08383 HMDB08286	PC 42:5 e	0.339 (0.292)	−0.145 (0.333)	0.0064	0.0364	Down
NA	LPI 14:0	0.660 (0.510)	−0.283 (0.689)	0.0077	0.0432	Down
HMDB06547	Stearidonic acid-iso1	−0.405 (0.284)	0.173 (0.431)	0.0078	0.0432	Up
HMDB01383	Sphingosine-1-P	0.402 (0.341)	−0.172 (0.414)	0.0081	0.0440	Down
HMDB01032	Dehydro-epiandrost-erone sulfate	0.607 (0.361)	−0.260 (0.670)	0.0085	0.0440	Down
HMDB00123	Glycine	−0.260 (0.085)	0.111 (0.305)	0.0087	0.0440	Up
HMDB00575	Homo-cystine	−0.472 (0.373)	0.202 (0.649)	0.0087	0.0440	Up
HMDB05461	TG 54:5	−0.324 (0.164)	0.139 (0.614)	0.0087	0.0440	Up
HMDB02802	Cortisone	0.715 (0.138)	−0.307 (0.574)	0.0087	0.0440	Down
HMDB02226	Adrenic acid	−0.421 (0.335)	0.180 (0.447)	0.0087	0.0440	Up
HMDB00112	gamma-Aminobutyric acid	−0.449 (0.609)	0.193 (0.373)	0.0092	0.0460	Up
HMDB00162	Proline	−0.308 (0.256)	0.132 (0.329)	0.0096	0.0471	Up
HMDB00673	Linolenic acid-iso1	−0.414 (0.387)	0.178 (0.432)	0.0098	0.0474	Up
HMDB10391	LPC 20:1-sn2	0.526 (0.403)	−0.225 (0.578)	0.0100	0.0480	Down
NA	LPC 22:3-sn2	0.498 (0.458)	−0.214 (0.530)	0.0105	0.0497	Down
NA	LPI 16:0	0.577 (0.324)	−0.247 (0.668)	0.0107	0.0498	Down

DG, diglycerides; ChoE, cholesteryl ester; HDHA, hydroxy-docosahexaenoic acid; LPE, lyso-phosphatidylethanolamine; LPI, lyso-phosphatidylinositol; LPC, lyso-phosphatidylcholines; PC, phosphatidylcholines; TG, triglycerides.

**Table 3:** Over representation analysis of different metabolites in preeclampsia vs controls based on the Kyoto Encyclopedia of Genes and Genome (KEGG).

Pathway	Total	Expected	Hits	Raw p	−log(10)p	Adjusted p	FDR	Impact
Aminoacyl-tRNA biosynthesis	48	17.03	16	0.0006 × 10^−9^	12.20	0.05 × 10^−9^	0.05 × 10^−9^	0
Arginine biosynthesis	14	0.49	6	0.004 × 10^−3^	54.37	0.0003	0.0002	0.48
Alanine, aspartate and glutamate metabolism	28	0.99	7	0.03 × 10^−3^	44.95	0.003	0.0009	0.45
Valine, leucine and isoleucine biosynthesis	8	0.28	4	0.09 × 10^−3^	40.48	0.007	0.002	0
Linoleic acid metabolism	5	0.18	3	0.0004	33.95	0.032	0.007	1
D-Glutamine and D-glutamate metabolism	6	0.21	3	0.0008	31.05	0.06	0.011	0.50
Arginine and proline metabolism	38	13.48	6	0.0017	27.60	0.14	0.020	0.36
Histidine metabolism	16	0.57	4	0.0018	27.25	0.14	0.020	0.27
Phenylalanine, tyrosine and tryptophan biosynthesis	4	0.14	2	0.007	21.50	0.54	0.07	1
Butanoate metabolism	15	0.53	3	0.014	18.47	1	0.12	0.03
Nitrogen metabolism	6	0.21	2	0.017	17.72	1	0.13	0
Glyoxylate and dicarboxylate metabolism	32	11.36	4	0.024	1.61	1	0.17	0.11
Glycine, serine and threonine metabolism	33	1.17	4	0.027	15.68	1	0.17	0.34
Phenylalanine metabolism	10	0.35	2	0.05	13.34	1	0.28	0.36
Lysine degradation	25	0.89	3	0.06	1.25	1	0.31	0.14
Glutathione metabolism	28	0.99	3	0.07	11.31	1	0.37	0.11
alpha-Linolenic acid metabolism	13	0.46	2	0.08	11.24	1	0.37	0
Cysteine and methionine metabolism	33	1.17	3	0.11	0.96	1	0.51	0.10
Glycerophospholipid metabolism	36	12.77	3	0.13	0.88	1	0.59	0.13
Citrate cycle (TCA cycle)	20	0.71	2	0.16	0.81	1	0.65	0.10
Valine, leucine and isoleucine degradation	40	14.19	3	0.17	0.78	1	0.65	0
beta-Alanine metabolism	21	0.75	2	0.17	0.77	1	0.65	0
Pyruvate metabolism	22	0.78	2	0.18	0.74	1	0.67	0.29
Ubiquinone and other terpenoid-quinone biosynthesis	9	0.32	1	0.28	0.56	1	0.97	0
Porphyrin and chlorophyll metabolism	30	10.65	2	0.29	0.54	1	0.97	0
Biotin metabolism	10	0.35	1	0.30	0.52	1	0.98	0
Steroid hormone biosynthesis	85	30.16	4	0.36	0.45	1	1	0.08
Glycero-lipid metabolism	16	0.57	1	0.44	0.36	1	1	0.01
Tyrosine metabolism	42	14.90	2	0.44	0.35	1	1	0.14
Primary bile acid biosynthesis	46	16.32	2	0.49	0.31	1	1	0.02
Pantothenate and CoA biosynthesis	19	0.67	1	0.50	0.30	1	1	0
Seleno-compound metabolism	20	0.71	1	0.52	0.29	1	1	0
Sphingolipid metabolism	21	0.75	1	0.53	0.27	1	1	0.02
Propanoate metabolism	23	0.82	1	0.57	0.25	1	1	0
Glycolysis/Gluconeogenesis	26	0.92	1	0.61	0.21	1	1	0.10
Arachidonic acid metabolism	36	12.77	1	0.73	0.14	1	1	0
Biosynthesis of unsaturated fatty acids	36	12.77	1	0.73	0.14	1	1	0
Pyrimidine metabolism	39	13.84	1	0.76	0.12	1	1	0
Steroid biosynthesis	42	14.90	1	0.79	0.11	1	1	0
Purine metabolism	65	23.07	1	0.91	0.04	1	1	0

## Data Availability

The metabolomics data reported in this study are available as supplementary information.

## Supplementary Materials

The following are available online at https://www.mdpi.com/article/10.3390/life12010086/s1, Table S1: Univariate analysis results for each metabolite.

## Appendix A

### Appendix A.1. Methods

#### Appendix A.1.1. Metabolomics Technique

##### Method 1: Lipidomic Analysis with Methanol Extraction

For the extraction of less hydrophobic lipids, a protein precipitation extraction was performed by adding four volumes of methanol containing internal standard mixture (myristic acid-d27, arachidonic acid-d8, Cholic acid-d5, Taurocholic acid-d5 LysoPC 18:1-d7, and labelled Carnitine Mix from Cambridge Isotopes) to serum. Then, the samples were mixed and incubated at −20 °C for 30 min., centrifuged at 15,000 rpm and supernatant was analysed by UHPLC-qTOF (model 6550 of Agilent, Agilent, Santa Clara, CA USA) in both positive and negative electrospray ionization mode.

The chromatographic method was the same for both ionization modes. A gradient elution with water and acetonitrile with 0.05% formic acid as mobile phase and C18 column (ACQUITY UPLC BEH C18 Column, 1.7 μm, 2.1 mm × 100 mm) that allowed the sequential elution of the less hydrophobic lipids such as non-esterified fatty acids, acyl carnitines, bile acids, steroids and lysophospholipids among others.

The identification of lipid species was performed by matching their accurate mass and tandem mass spectrum, when available, to Metlin-PCDL from Agilent containing more than 40,000 metabolites and lipids. In addition, chromatographic behavior of pure standards for each family and bibliographic information was used to ensure their putative identification.

After putative identification of lipids, these were semi quantified depending on their family similarity by an internal standard calibration curves using the pure chemical standards indicated in Table A1.

**Table A1:** Standards used for methanol lipidomic extraction.

Standards	
15-HETE	lysoPC 18:1
arachidonic acid	lysoPE18:1
Cholic acid	lysoPI 18:1
Deoxycholic acid	sphingosine-1-P
Dehydroisoandosterone-3-sulfate	Taurocholic acid
Hydrocortisone (cortisol)	Carnitine Mix

##### Method 2: Lipidomic Analysis with Choloform:Methanol (Folch Method) Extraction

For the extraction of more hydrophobic lipids, a liquid-liquid extraction with chloroform:methanol (2:1) based on Folch procedure was performed by adding four volumes of chloroform:methanol (2:1) containing internal standard mixture (Lipidomic SPLASH®, Avanti Polar Lipids, Birmingham, AL, USA) to serum. Then, the samples were mixed and incubated at −20 °C for 30 min. Afterwards, water with NaCl (0.8%) was added and mixture was centrifuged at 15,000 rpm. Lower phase was recovered, evaporated to dryness and reconstituted with methanol:methyl-*tert*-butyl ether (9:1) and analysed by UHPLC-qTOF (model 6550 of Agilent, USA) in both positive and negative electrospray ionization modes.

As method 1, the chromatographic method was the same for both ionizations. Gradient consist in an elution with a ternary mobile phase containing water, methanol and 2-propanol with 10mM ammonium formate and 0.1% formic acid. The stationary phase was a C18 column (Kinetex EVO C18 Column, 2.6 μm, 2.1 mm × 100 mm) that allows the sequential elution of the more hydrophobic lipids such as lysophospholipids, sphingomyelins, phospholipids, diglycerides, triglycerides and cholesteryl esters, among others.

The identification of lipid species was performed by matching their accurate mass and tandem mass spectrum, when available, to Metlin-PCDL from Agilent containing more than 40,000 metabolites and lipids. In addition, chromatographic behaviour of pure standards for each family and bibliographic information was used to ensure their putative identification.

After putative identification of lipids, these were semi quantified depending on their family similarity by and internal standard calibration curves using the pure chemical standards indicated in Table A2.

**Table A2:** Standards used for Folch based lipidomic analysis.

Standards
Sphingomyelin d18:1/16:0
Stearoyl-rac-glycerol
Oelamide
Dilynoleyl-rac-glycerol
Palmitoyl-oleyl-lynoleyl-rac-glycerol
Dipalmitoyl-phosphoethanolamine
Dioleyl-phosphocholine
Cholesteryl linoleate

##### Method 3: Aminoacids Analysis

For the extraction of aminoacids, a protein precipitation extraction was performed by adding four volumes of methanol containing internal standards (Metabolomics labelled aminoacid mixture from Cambridge Isotopes) to serum samples. Then, the samples were mixed and incubated at −20 °C for 30 min., centrifuged at 15,000 rpm and supernatant was derivatized using AccQ-Tag reagent from Waters® (Milford, MA, USA) following manufacturing protocol. Then, derivatized aminoacids were analysed by UHPLC-QqQ (model 6490 of Agilent, USA) in Multiple Reaction Monitoring acquisition.

The chromatographic separation consists of a gradient elution with water and acetonitrile with 0.1% formic acid as mobile phase and C18 column (ACQUITY UPLC HSS T3 Column, 1.7 μm, 2.1 mm × 150 mm) that allows the determination and separation of aminoacids and derivatives. Their semi-quantification was done with pure chemical standard curves for all compounds.

##### Method 4: Polar Metabolites and Central Carbon Metabolism

For the extraction of polar metabolites and central carbon metabolism, a protein precipitation extraction was performed by adding eight volumes of methanol:water (8:2) containing internal standard mixture (succinic acid-d4, myristic acid-d27, glicerol-13C3 and D-glucose-13C6) to serum samples. Then, the samples were mixed and incubated at 4ºC for 10 min., centrifuged at 15,000 rpm and supernatant was evaporated to dryness and freeze dried in a liophylizator before compound derivatization (metoximation and silylation). The derivatized compounds were analysed by GC-qTOF (model 7200 of Agilent, USA).

The chromatographic separation was based on Fiehn Method, using a J&W Scientific HP5-MS (30 m × 0.25 mm i.d., 0.25 μm film capillary column and helium as carrier gas using an oven program from 60 to 325 °C. Ionization was done by electronic impact (EI), with electron energy of 70 eV and operated in full Scan mode.

In addition of targeted compounds from central carbon metabolism, a screening for the identification of more metabolites was performed by matching their EI mass spectrum and retention time to metabolomic Fiehn library (from Agilent) which contains more than 1.400 metabolites.

After putative identification of metabolites, these and target compounds were semi-quantified depending on their family similarity by an internal standard calibration curves using the pure chemical standards indicated in Table A3.

**Table A3:** Standards used for central carbon metabolism analysis.

Compound	
Piruvic acid	Aconitic acid
Lactic acid	3-Phosphoglyceric acid
Glycolic acid	Citric/isocitric acid
2-Hydroxybutyric acid	Myristic acid
Oxalic Acid	Glucose
3-Hydroxybutiric acid	Palmitic acid
Glycerol	Ribose-5-phosphate
Succinic acid	Linoleic acid
Glyceric acid	Oleic Acid
Fumaric acid	Stearic acid
Malic acid	Fructose-6-phosphate
Threonic acid	Glucose-6-phosphate
2-hydroxyglutaric acid	Fructose-1,6-bisphosphate
alpha-Ketoglutaric acid	6-Phosphogluconic acid
Phosphoenolpyruvate	Sucrose
Ornithine	alpha-Tocopherol
Glyceraldehyde-3-phosphate	Cholesterol

### Appendix A.2. Statistical Analysis

For metabolomics data, statistical approach was performed using Metaboanalyst 4.0 (http://www.metaboanalyst.ca/, accessed on 11 August 2021). Only those metabolites that were present in ≥ 70% of the samples in at least one group were considered. To estimate missing values in the included metabolites, we used a Bayesian principal component analysis approach. Then, a log base 2 transformation was applied, and data were mean-centered for univariate analyses and Pareto scaled for multivariate analyses. Initially, a multivariate modelling was performed including the use of unsupervised methods such as principal component analysis (PCA), and supervised methods like partial least squares discriminant analysis (PLS-DA) and an orthogonal projection to latent structures discriminant analysis (OPLS-DA). A variable importance in projection (VIP) plot, which is a visual representation of the importance of the particular metabolites in discriminating the groups of interest, is provided. To assess the significance of class discrimination whether it could be due to chance, a permutation test was performed applying 2000 permutations. For the selected model, the goodness of fit (R2X) and the predictive performance (Q2Y), which relate to the explained and predicted variance respectively, were calculated. All these methods were applied with a Pareto scaling.

Secondly, for each metabolite a univariate Student’s *t*-test was performed and Benjamini-Hochberg method was used to adjust p values for multiple testing with consideration of 5% false discovery rate. An additional unsupervised hierarchical clustering analysis (HCA) was performed based on the univariate results.

Last, differential pathways were identified from an over representation analysis (ORA) performed in metaboanalyst using the Metabolite Set Enrichment Analysis option. Significantly different metabolites based on the univariate analysis were used for the pathway analyses. The ORA is to test if certain groups of metabolites are represented more often than expected by chance within a given metabolite list. This was tested statistically using a Fisher’s exact test to calculate the probability of seeing at least a particular number of metabolites containing a biological term of interest in the given compound list.
