# Supplementary material for: The Interplay between Pathophysiological Pathways in Early-Onset Severe Preeclampsia Unveiled by Metabolomics
Source: Life (Basel). 2022 Jan 7;12(1):86. doi: 10.3390/life12010086 (PMC8780941; doi:10.3390/life12010086)
Supplement: Supplementary file 1 [file life-12-00086-s001.zip › life-1527069-supplementary.pdf]

life

MDPI

Supplementary Materials

Table S1. Univariate analysis results for each metabolite. *p*–value is calculated with t–test as a default. *p*–value with (W) is calculated by the Wilcoxon Mann Whitney test. q–value is calculated after 5% false discovery rate correction.

Metabolite Name	Controls Mean (SD)	Preeclampsia Mean (SD)	*p*–Value	*q*–Value	Preeclampsia/ Controls
Cystine	−0.553 (0.167)	0.237 (0.251)	< 0.0001	< 0.0001	Up
Ethanolamine	−0.397 (0.160)	0.170 (0.174)	< 0.0001	< 0.0001	Up
Tyrosine	−0.431 (0.070)	0.185 (0.238)	< 0.0001	< 0.0001	Up
Free carnitine	−0.572 (0.138)	0.245 (0.393)	< 0.0001	< 0.0001	Up
Anserine	−0.361 (0.130)	0.155 (0.183)	< 0.0001	0.0006	Up
Propionyl–carnitine	−0.581 (0.079)	0.249 (0.464)	< 0.0001	0.0006	Up
Isobutyryl–carnitine/Butyryl–carnitine	−0.565 (0.120)	0.242 (0.465)	< 0.0001	0.0011	Up
Glycocholic acid–iso3	0.819 (0.401)	−0.351 (0.450)	< 0.0001	0.0014	Down
Homocitrulline	−0.614 (0.158)	0.263 (0.318)	< 0.0001 (W)	0.0021	Up
Pyruvic acid	−0.505 (0.282)	0.216 (0.300)	< 0.0001	0.0034	Up
Pregnenolone sulfate	0.708 (0.395)	−0.303 (0.426)	0.0001	0.0035	Down
Methionine	−0.512 (0.144)	0.219 (0.351)	0.0001	0.0038	Up
L–2–Aminoadipic acid	−0.561 (0.334)	0.240 (0.339)	0.0001	0.0038	Up
Linolenic acid–iso2	−0.567 (0.368)	0.243 (0.341)	0.0002	0.0044	Up
Hydroxylysine	−0.490 (0.292)	0.210 (0.354)	0.0002 (W)	0.0054	Up
2–methyl–butyryl–carnitine/Isovaleryl–carnitine/Valeryl–carnitine	−0.562 (0.219)	0.241 (0.403)	0.0002	0.0060	Up
Glycocholic acid–iso2	0.719 (0.650)	−0.308 (0.374)	0.0003	0.0061	Down
1–Methylhistidine	−0.524 (0.168)	0.224 (0.390)	0.0003	0.0064	Up
Lysine	−0.374 (0.181)	0.160 (0.271)	0.0004	0.0069	Up
Stearidonic acid	−0.650 (0.508)	0.278 (0.534)	0.0004 (W)	0.0069	Up
Citrulline	−0.300 (0.080)	0.129 (0.341)	0.0004	0.0075	Up
Glutamine	−0.396 (0.169)	0.170 (0.301)	0.0005	0.0075	Up
LPE 20:2–sn1	0.616 (0.513)	−0.264 (0.380)	0.0005	0.0075	Down
Cortisol	0.979 (0.168)	−0.420 (0.561)	0.0006 (W)	0.0089	Down
Asparagine	−0.311 (0.127)	0.133 (0.352)	0.0006	0.0089	Up
Histidine	−0.372 (0.132)	0.160 (0.299)	0.0006	0.0089	Up
Testosterone	1.018 (0.787)	−0.436 (0.695)	0.0006	0.0089	Down
Glycocholic acid–iso1	0.716 (0.543)	−0.307 (0.500)	0.0007	0.0094	Down
Leucine	−0.376 (0.195)	0.161 (0.294)	0.0007	0.0096	Up
2–Hydroxybutyric acid	−0.611 (0.395)	0.262 (0.460)	0.0008	0.0098	Up
LPI 18:1	0.631 (0.252)	−0.271 (0.740)	0.0008	0.0098	Down
Phenylalanine	−0.366 (0.170)	0.157 (0.298)	0.0009	0.0104	Up
LPC 16:1 e	−0.294 (0.166)	0.126 (0.235)	0.0009	0.0108	Up
alpha–Ketoglutaric acid	−0.368 (0.208)	0.158 (0.306)	0.0013	0.0139	Up
11.13–Eicosadienoic acid	−0.530 (0.377)	0.227 (0.418)	0.0013	0.0139	Up
Arginine	−0.297 (0.132)	0.127 (0.367)	0.0013	0.0139	Up
17–HDHA	−0.648 (0.384)	0.278 (0.443)	0.0013 (W)	0.0139	Up
Threonine	−0.415 (0.295)	0.178 (0.353)	0.0015 (W)	0.0156	Up
Valine	−0.344 (0.265)	0.147 (0.274)	0.0016	0.0157	Up
w3–docosapentaenoic acid	−0.524 (0.416)	0.225 (0.435)	0.0022	0.0203	Up

Isoleucine	−0.309 (0.123)	0.133 (0.289)	0.0022	0.0203	Up
Glutamic acid	−0.262 (0.267)	0.112 (0.191)	0.0022	0.0203	Up
Ornithine	−0.439 (0.261)	0.188 (0.394)	0.0023	0.0203	Up
Ursodeoxycholic/Hyodeoxicholic acid	0.650 (0.394)	−0.279 (0.584)	0.0024	0.0204	Down
DG 34:3	−0.411 (0.335)	0.176 (0.344)	0.0024	0.0204	Up
Lactic acid	−0.295 (0.151)	0.126 (0.273)	0.0024	0.0204	Up
pentadecanoic acid	−0.440 (0.236)	0.189 (0.407)	0.0025	0.0206	Up
TG 54:6	−0.517 (0.475)	0.221 (0.415)	0.0026	0.0206	Up
Eicosenoic acid	−0.521 (0.332)	0.223 (0.474)	0.0027	0.0213	Up
Sarcosine	−0.481 (0.338)	0.206 (0.436)	0.0030	0.0230	Up
Alanine	−0.378 (0.185)	0.162 (0.367)	0.0033	0.0243	Up
trans−palmitoleic acid palmitelaidic	−0.497 (0.244)	0.213 (0.607)	0.0033 (W)	0.0243	Up
ChoE 20:2	0.320 (0.336)	−0.137 (0.252)	0.0034 (W)	0.0243	Down
LPC 22:3−sn1	0.629 (0.343)	−0.269 (0.607)	0.0034	0.0243	Down
TG 54:7	−0.543 (0.565)	0.233 (0.433)	0.0035	0.0243	Up
PC 34:1 e	0.350 (0.286)	−0.150 (0.315)	0.0037	0.0249	Down
LPI 18:2	0.624 (0.343)	−0.267 (0.609)	0.0037	0.0249	Down
12,13−EpOME9	−0.461 (0.293)	0.198 (0.443)	0.0039	0.0254	Up
LPC 20:2−sn2	0.516 (0.470)	−0.221 (0.455)	0.0041	0.0264	Down
LPI 18:0	0.633 (0.280)	−0.271 (0.643)	0.0042	0.0267	Down
3−Aminobutanoic acid	−0.415 (0.227)	0.178 (0.414)	0.0042	0.0267	Up
HODE−iso1	−0.512 (0.440)	0.219 (0.475)	0.0048	0.0295	Up
LPI 22:4	0.548 (0.358)	−0.235 (0.546)	0.0049	0.0300	Down
LPI 20:4	0.590 (0.305)	−0.253 (0.613)	0.0053	0.0320	Down
LPC 18:2−sn2	0.453 (0.348)	−0.194 (0.446)	0.0056	0.0330	Down
2−hydroxyglutaric acid	−0.346 (0.099)	0.148 (0.557)	0.0060	0.0349	Up
PC 42:5 e	0.339 (0.292)	−0.145 (0.333)	0.0064	0.0364	Down
LPI 14:0	0.660 (0.510)	−0.283 (0.689)	0.0077	0.0432	Down
Stearidonic acid−iso1	−0.405 (0.284)	0.173 (0.431)	0.0078	0.0432	Up
Sphingosine−1−P	0.402 (0.341)	−0.172 (0.414)	0.0081	0.0440	Down
Dehydroepiandrosterone sulfate	0.607 (0.361)	−0.260 (0.670)	0.0085	0.0440	Down
Glycine	−0.260 (0.085)	0.111 (0.305)	0.0087 (W)	0.0440	Up
Homocystine	−0.472 (0.373)	0.202 (0.649)	0.0087 (W)	0.0440	Up
TG 54:5	−0.324 (0.164)	0.139 (0.614)	0.0087 (W)	0.0440	Up
Cortisone	0.715 (0.138)	−0.307 (0.574)	0.0087 (W)	0.0440	Down
Adrenic acid	−0.421 (0.335)	0.180 (0.447)	0.0087	0.0440	Up
gamma−Aminobutyric acid	−0.449 (0.609)	0.193 (0.373)	0.0092	0.0460	Up
Proline	−0.308 (0.256)	0.132 (0.329)	0.0096	0.0471	Up
Linolenic acid−iso1	−0.414 (0.387)	0.178 (0.432)	0.0098	0.0474	Up
LPC 20:1−sn2	0.526 (0.403)	−0.225 (0.578)	0.0100	0.0480	Down
LPC 22:3−sn2	0.498 (0.458)	−0.214 (0.530)	0.0105	0.0497	Down
LPI 16:0	0.577 (0.324)	−0.247 (0.668)	0.0107	0.0498	Down
Sphinganine−1−P	0.417 (0.404)	−0.179 (0.441)	0.0111	0.0512	Down
Linoleic acid−iso2	−0.359 (0.346)	0.154 (0.382)	0.0113	0.0516	Up
Cystathione	−0.527 (0.740)	0.226 (0.453)	0.0114	0.0516	Up
Myristic acid	−0.383 (0.259)	0.164 (0.443)	0.0118	0.0521	Up
Hexanoyl−carnitine	−0.410 (0.291)	0.176 (0.472)	0.0118	0.0521	Up

LPC 20:1−sn1	0.528 (0.428)	−0.226 (0.596)	0.0121	0.0527	Down
TG 52:6	−0.372 (0.477)	0.160 (0.353)	0.0123	0.0528	Up
LPC 15:0−sn1 d	0.458 (0.389)	−0.196 (0.514)	0.0125	0.0531	Down
LPC 19:1−sn1	0.546 (0.448)	−0.234 (0.619)	0.0126	0.0531	Down
SM 41:2	0.270 (0.264)	−0.116 (0.299)	0.0138	0.0576	Down
ChoE 16:0	0.242 (0.301)	−0.104 (0.247)	0.0149	0.0609	Down
LPC 17:0−sn1	0.477 (0.428)	−0.204 (0.550)	0.0149	0.0609	Down
LPI 17:0	0.570 (0.373)	−0.244 (0.694)	0.0151	0.0610	Down
PC 40:4 e	0.288 (0.229)	−0.123 (0.342)	0.0154	0.0614	Down
Octenoyl−carnitine	−0.432 (0.300)	0.185 (0.527)	0.0158	0.0614	Up
LPC 19:0−sn1	0.488 (0.383)	−0.209 (0.585)	0.0159	0.0614	Down
Linoleic acid−iso1	−0.387 (0.320)	0.166 (0.460)	0.0160	0.0614	Up
PC 36:2 e	0.380 (0.481)	−0.163 (0.392)	0.0160	0.0614	Down
Serine	−0.277 (0.177)	0.119 (0.347)	0.0172	0.0653	Up
LPC 16:0−sn2	0.394 (0.324)	−0.169 (0.478)	0.0174	0.0653	Down
LPC 18:0−sn2	0.451 (0.334)	−0.193 (0.562)	0.0185	0.0687	Down
LPC 18:1−sn2	0.415 (0.334)	−0.178 (0.516)	0.0192	0.0707	Down
androsterone sulfate−iso1	0.494 (0.300)	−0.212 (0.642)	0.0200 (W)	0.0730	Down
Sucrose	−0.470 (0.312)	0.201 (0.608)	0.0206	0.0739	Up
Cholesterol	0.238 (0.218)	−0.102 (0.294)	0.0208	0.0739	Down
LPC 18:0 e	0.314 (0.329)	−0.135 (0.346)	0.0208 (W)	0.0739	Down
PC 38:4 e	0.326 (0.359)	−0.140 (0.386)	0.0213	0.0747	Down
LPC 19:0−sn2	0.448 (0.431)	−0.192 (0.552)	0.0215	0.0747	Down
DG 34:2	−0.363 (0.419)	0.155 (0.425)	0.0219	0.0757	Up
LPC 15:0−sn2	0.449 (0.447)	−0.193 (0.560)	0.0234	0.0800	Down
LPC 14:0−sn1	0.464 (0.468)	−0.199 (0.601)	0.0276	0.0937	Down
LPI 20:3	0.478 (0.337)	−0.205 (0.673)	0.0312	0.1047	Down
LPC 22:4−sn1	0.442 (0.408)	−0.189 (0.605)	0.0323	0.1066	Down
AIBA	−0.276 (0.121)	0.118 (0.511)	0.0326 (W)	0.1066	Up
SM 43:1	0.257 (0.317)	−0.110 (0.307)	0.0326 (W)	0.1066	Down
Androstenedione	0.503 (0.705)	−0.216 (0.610)	0.0329	0.1068	Down
PC 36:0	0.239 (0.279)	−0.102 (0.313)	0.0336	0.1082	Down
Glycoursodeoxycholic acid	0.502 (0.874)	−0.215 (0.526)	0.0342	0.1089	Down
LPC 14:0−sn2	0.461 (0.495)	−0.197 (0.622)	0.0344	0.1089	Down
OxoODE−iso1	−0.264 (0.286)	0.113 (0.487)	0.0353 (W)	0.1104	Up
PC 40:5 e	0.265 (0.251)	−0.114 (0.370)	0.0355	0.1104	Down
LPC 20:0−sn2	0.423 (0.358)	−0.181 (0.603)	0.0359	0.1107	Down
Hexadecanoyl−carnitine	−0.272 (0.303)	0.117 (0.369)	0.0361	0.1107	Up
TG 52:5	−0.324 (0.422)	0.139 (0.421)	0.0373	0.1135	Up
LPC 20:3−sn2	0.413 (0.420)	−0.177 (0.581)	0.0382	0.1153	Down
cis−palmitoleic acid palmitoleic	−0.386 (0.612)	0.165 (0.464)	0.0397	0.1188	Up
LPI 22:6	0.488 (0.384)	−0.209 (0.722)	0.0404	0.1200	Down
PC 38:5 e	0.242 (0.246)	−0.104 (0.350)	0.0421	0.1240	Down
LPC 18:3−sn2	0.478 (0.520)	−0.205 (0.688)	0.0438	0.1274	Down
LPE 18:1−sn1	0.388 (0.484)	−0.166 (0.538)	0.0439	0.1274	Down
LPE 20:1−sn1	0.382 (0.407)	−0.164 (0.563)	0.0469	0.1349	Down
LPC 18:3−sn1	0.453 (0.457)	−0.194 (0.676)	0.0473	0.1351	Down
LPE 20:4−sn2	0.371 (0.434)	−0.159 (0.541)	0.0484	0.1373	Down
N−palmitoylethanolamine	−0.247 (0.161)	0.106 (0.390)	0.0490	0.1379	Up

LPE 17:0−sn2	0.428 (0.591)	−0.184 (0.605)	0.0515	0.1440	Down
LPE 14:0−sn2	0.388 (0.581)	−0.166 (0.542)	0.0552	0.1523	Down
LPE 20:4−sn1	0.374 (0.394)	−0.160 (0.580)	0.0553	0.1523	Down
Oxalic acid	0.223 (0.228)	−0.096 (0.349)	0.0561	0.1534	Down
Oleic acid	−0.278 (0.429)	0.119 (0.390)	0.0573	0.1556	Up
LPI 16:1	0.489 (0.518)	−0.210 (0.766)	0.0577	0.1556	Down
PC 38:3 e	0.333 (0.308)	−0.143 (0.528)	0.0622 (W)	0.1654	Down
PC 42:4 e	0.198 (0.340)	−0.085 (0.382)	0.0622 (W)	0.1654	Down
LPC 20:4−sn2	0.339 (0.266)	−0.145 (0.571)	0.0651	0.1703	Down
LPI 20:5	0.497 (0.451)	−0.213 (0.827)	0.0654	0.1703	Down
Palmitic acid	−0.233 (0.301)	0.100 (0.365)	0.0657	0.1703	Up
LPE 18:2−sn1	0.332 (0.557)	−0.142 (0.471)	0.0658	0.1703	Down
LPC 22:6−sn1	0.357 (0.331)	−0.153 (0.601)	0.0688	0.1769	Down
LPC 17:1−sn1	0.344 (0.342)	−0.148 (0.585)	0.0728	0.1851	Down
DG 34:1	−0.283 (0.445)	0.121 (0.431)	0.0730	0.1851	Up
Glucose	−0.214 (0.377)	0.092 (0.313)	0.0757	0.1906	Up
Hydroxyproline trans	−0.165 (0.199)	0.071 (0.276)	0.0769	0.1925	Up
N−oleoyl ethanolamine	−0.219 (0.199)	0.094 (0.390)	0.0813	0.2021	Up
LPE 14:0−sn1	0.364 (0.599)	−0.156 (0.572)	0.0824	0.2030	Down
Decanoyl−carnitine/Fumaryl−carnitine	0.279 (0.428)	−0.120 (0.452)	0.0834	0.2030	Down
SM 41:1	0.179 (0.291)	−0.077 (0.284)	0.0837	0.2030	Down
Glycocholic acid−iso4	0.484 (0.629)	−0.208 (0.823)	0.0837	0.2030	Down
LPC 22:4−sn2	0.357 (0.393)	−0.153 (0.631)	0.0860	0.2072	Down
Octadecadienoyl−carnitine	−0.281 (0.494)	0.120 (0.440)	0.0874	0.2092	Up
TG 48:1	−0.261 (0.557)	0.112 (0.543)	0.0913 (W)	0.2159	Up
Taurocholic acid	−0.507 (0.924)	0.217 (0.749)	0.0913 (W)	0.2159	Up
Nonadecenoic acid	0.303 (0.681)	−0.130 (0.410)	0.0928	0.2181	Down
Stearic acid	−0.250 (0.503)	0.107 (0.377)	0.0958	0.2236	Up
ChoE 18:1	0.193 (0.372)	−0.083 (0.301)	0.0963	0.2236	Down
LPE 20:1−sn2	0.327 (0.478)	−0.140 (0.572)	0.0974	0.2247	Down
LPC 16:1−sn2	0.357 (0.487)	−0.153 (0.638)	0.0985	0.2259	Down
PC 36:3 e	0.298 (0.337)	−0.128 (0.555)	0.1009	0.2299	Down
13−OxoODE	−0.310 (0.592)	0.133 (0.511)	0.1073	0.2389	Up
SM 40:2	0.191 (0.238)	−0.082 (0.359)	0.1084	0.2389	Down
Taurochenodeoxycholic acid	−0.432 (1.008)	0.185 (0.622)	0.1084	0.2389	Up
SM 39:1	0.221 (0.358)	−0.095 (0.322)	0.1093 (W)	0.2389	Down
TG 50:0	−0.428 (0.438)	0.183 (0.735)	0.1093 (W)	0.2389	Up
TG 50:2	−0.246 (0.384)	0.105 (0.474)	0.1093 (W)	0.2389	Up
ChoE 20:4	0.175 (0.199)	−0.075 (0.338)	0.1108	0.2389	Down
Docosahexaenoic acid DHA	−0.235 (0.216)	0.101 (0.463)	0.1108	0.2389	Up
LPE 18:2−sn2	0.282 (0.588)	−0.121 (0.450)	0.1110	0.2389	Down
9,10−EpOME12	−0.364 (0.593)	0.156 (0.651)	0.1111	0.2389	Up
w6−docosapentaenoic acid	−0.246 (0.235)	0.106 (0.486)	0.1116	0.2389	Up
Citric/isocitric acid	−0.168 (0.132)	0.072 (0.501)	0.1148	0.2443	Up
DG 36:4	−0.275 (0.508)	0.118 (0.488)	0.1204	0.2548	Up
ChoE 20:3	0.216 (0.280)	−0.093 (0.424)	0.1215	0.2557	Down
TG 48:0	−0.309 (0.569)	0.133 (0.561)	0.1249	0.2613	Up
LPC 16:1−sn1	0.335 (0.498)	−0.144 (0.649)	0.1260	0.2623	Down

TG 48:2	−0.242 (0.487)	0.104 (0.533)	0.1297 (W)	0.2670	Up
LPC 17:0−sn2	0.466 (0.725)	−0.200 (0.783)	0.1297 (W)	0.2670	Down
LPE 22:4−sn1	0.316 (0.577)	−0.135 (0.589)	0.1316	0.2696	Down
HETE−iso1	−0.200 (0.268)	0.086 (0.498)	0.1372 (W)	0.2788	Up
Octadecenoyl−carnitine	−0.241 (0.294)	0.103 (0.503)	0.1377	0.2788	Up
LPE 15:0−sn1	0.311 (0.565)	−0.133 (0.597)	0.1384	0.2788	Down
TG 50:3	−0.250 (0.446)	0.107 (0.482)	0.1390	0.2788	Up
Deoxycholic acid−iso2	0.322 (0.547)	−0.138 (0.636)	0.1411	0.2814	Down
LPE 15:0−sn2	0.298 (0.608)	−0.128 (0.554)	0.1423	0.2823	Down
LPE 20:3−sn1	0.312 (0.606)	−0.134 (0.603)	0.1482	0.2926	Down
Acetyl−carnitine	−0.247 (0.329)	0.106 (0.528)	0.1507	0.2960	Up
dihomo−gamma−linolenic acid−iso3	−0.097 (0.282)	0.042 (0.483)	0.1528 (W)	0.2987	Up
14,15−DiHETE	−0.339 (0.415)	0.145 (0.741)	0.1537	0.2988	Up
TG 50:4	−0.230 (0.484)	0.098 (0.440)	0.1549	0.2996	Up
Deoxycholic acid−iso1	0.440 (0.903)	−0.189 (0.464)	0.1557	0.2996	Down
LPE 22:5−sn2	0.329 (0.646)	−0.141 (0.659)	0.1587	0.3029	Down
LPC 20:3−sn1	−0.202 (0.223)	0.087 (0.453)	0.1590	0.3029	Up
LPE 18:0−sn2	0.279 (0.499)	−0.119 (0.581)	0.1619	0.3069	Down
LPC 16:0 e	0.150 (0.204)	−0.064 (0.332)	0.1634	0.3077	Down
11,12−DiHETE	−0.374 (0.902)	0.160 (0.688)	0.1639	0.3077	Up
LPC 22:5−sn1	0.313 (0.571)	−0.134 (0.654)	0.1649	0.3080	Down
12−HETE	−0.325 (0.788)	0.139 (0.942)	0.1684 (W)	0.3125	Up
TG 50:1	−0.243 (0.468)	0.104 (0.507)	0.1689	0.3125	Up
LPE 22:4−sn2	0.271 (0.569)	−0.116 (0.552)	0.1718	0.3163	Down
9−OxoODE	−0.230 (0.377)	0.098 (0.557)	0.1734 (W)	0.3177	Up
SM 43:2	0.284 (0.419)	−0.122 (0.644)	0.1757	0.3202	Down
TG 46:0	−0.279 (0.577)	0.119 (0.581)	0.1764	0.3202	Up
LPE 16:1−sn1	0.280 (0.630)	−0.120 (0.569)	0.1790	0.3234	Down
LPE 18:0−sn1	0.261 (0.480)	−0.112 (0.572)	0.1806	0.3248	Down
LPE 16:0−sn1	0.257 (0.494)	−0.110 (0.565)	0.1859	0.3327	Down
TG 51:2	−0.215 (0.496)	0.092 (0.444)	0.1882	0.3353	Up
LPE 18:3−sn1	0.288 (0.534)	−0.123 (0.651)	0.1913	0.3392	Down
Taurodeoxycholic acid	−0.512 (1.176)	0.219 (0.476)	0.1935	0.3401	Up
eicosapentaenoic acid	−0.252 (0.394)	0.108 (0.594)	0.1939	0.3401	Up
androsterone sulfate−iso5	0.208 (0.556)	−0.089 (0.403)	0.1945	0.3401	Down
EpOME−iso1	−0.191 (0.406)	0.082 (0.428)	0.2004	0.3482	Up
Hexadecenyl−carnitine	−0.269 (0.541)	0.115 (0.536)	0.2009 (W)	0.3482	Up
PC 36:4 e	0.160 (0.253)	−0.068 (0.388)	0.2049	0.3535	Down
DG 36:3	−0.218 (0.460)	0.093 (0.497)	0.2068	0.3540	Up
PC 40:6	−0.181 (0.285)	0.078 (0.443)	0.2071	0.3540	Up
Malic acid	−0.136 (0.145)	0.058 (0.514)	0.2118	0.3589	Up
PC 32:1	−0.194 (0.473)	0.083 (0.425)	0.2122	0.3589	Up
SM 34:1	0.137 (0.305)	−0.059 (0.312)	0.2127	0.3589	Down
LPE 18:1−sn2	0.237 (0.644)	−0.102 (0.494)	0.2141	0.3596	Down
Tetradecanoyl−carnitine	−0.168 (0.359)	0.072 (0.537)	0.2155 (W)	0.3604	Up
LPE 17:1−sn1	0.250 (0.549)	−0.107 (0.584)	0.2190	0.3647	Down
PC 38:2	0.172 (0.265)	−0.074 (0.446)	0.2282	0.3770	Down
LPC 22:6−sn2	0.254 (0.373)	−0.109 (0.661)	0.2283	0.3770	Down

LPE 18:3−sn2	0.266 (0.612)	−0.114 (0.647)	0.2381	0.3880	Down
LPC 16:0−sn1	−0.126 (0.175)	0.054 (0.340)	0.2384	0.3880	Up
Tryptophan	0.064 (0.274)	−0.028 (0.288)	0.2391 (W)	0.3880	Down
SM 32:2	0.208 (0.266)	−0.089 (0.409)	0.2391 (W)	0.3880	Down
9,12,13−TriHOME10	−0.206 (0.212)	0.088 (0.490)	0.2403 (W)	0.3883	Up
ChoE 18:2	0.125 (0.246)	−0.054 (0.326)	0.2466	0.3968	Down
Kynurenine	0.120 (0.173)	−0.051 (0.471)	0.2500	0.4007	Down
LPC 20:5−sn1	0.301 (0.463)	−0.129 (0.849)	0.2632	0.4201	Down
SM 33:1	0.134 (0.296)	−0.058 (0.359)	0.2655	0.4214	Down
LPE 22:6−sn1	0.223 (0.534)	−0.096 (0.582)	0.2663	0.4214	Down
LPE 20:3−sn2	0.240 (0.618)	−0.103 (0.615)	0.2695	0.4247	Down
SM 42:3	0.127 (0.328)	−0.054 (0.330)	0.2739	0.4274	Down
Glycerol	−0.905 (3.041)	0.388 (0.199)	0.2740 (W)	0.4274	Up
SM 36:0	−0.177 (0.401)	0.076 (0.481)	0.2745	0.4274	Up
SM 42:1	0.121 (0.292)	−0.052 (0.324)	0.2767	0.4290	Down
SM 35:1	0.116 (0.330)	−0.050 (0.310)	0.2960	0.4545	Down
LPE 17:0−sn1	0.216 (0.556)	−0.093 (0.601)	0.2960	0.4545	Down
Methylmalonyl−carnitine/Succinyl −carnitine	0.224 (0.661)	−0.096 (0.251)	0.2966	0.4545	Down
LPE 16:0−sn2	0.213 (0.534)	−0.091 (0.606)	0.3024	0.4597	Down
Octanoyl−carnitine	0.161 (0.456)	−0.069 (0.439)	0.3032	0.4597	Down
15,16−epoxy−13−OH−9Z,11E−octad ecadienoic acid HpODE	0.176 (0.625)	−0.076 (0.422)	0.3036	0.4597	Down
LPE 17:1−sn2	0.197 (0.527)	−0.085 (0.556)	0.3058	0.4610	Down
LPC 18:2−sn1	−0.134 (0.434)	0.057 (0.348)	0.3080	0.4626	Up
Phosphoethanolamine	0.123 (0.225)	−0.053 (0.430)	0.3119 (W)	0.4666	Down
ChoE 17:1	0.152 (0.472)	−0.065 (0.423)	0.3225	0.4806	Down
LPC 17:1−sn2	0.202 (0.397)	−0.086 (0.659)	0.3361	0.4990	Down
LPC 20:5−sn2	0.261 (0.477)	−0.112 (0.863)	0.3382	0.5002	Down
LPC 22:5−sn2	0.186 (0.630)	−0.080 (0.530)	0.3432	0.5055	Down
TG 48:3	−0.168 (0.508)	0.072 (0.507)	0.3449	0.5062	Up
beta−Alanine	−0.126 (0.317)	0.054 (0.406)	0.3478	0.5064	Up
SM 32:1	0.126 (0.311)	−0.054 (0.408)	0.3483	0.5064	Down
TG 46:1	−0.188 (0.474)	0.080 (0.604)	0.3491	0.5064	Up
TG 52:2	−0.089 (0.366)	0.038 (0.499)	0.3530 (W)	0.5070	Up
LPC 20:0−sn1	0.118 (0.291)	−0.050 (0.385)	0.3535	0.5070	Down
SM 34:2	0.116 (0.262)	−0.050 (0.387)	0.3555	0.5070	Down
SM 40:1	0.109 (0.276)	−0.047 (0.359)	0.3560	0.5070	Down
DG 36:2	−0.146 (0.390)	0.063 (0.473)	0.3561	0.5070	Up
Tetradecadienoyl−carnitine	−0.193 (0.347)	0.083 (0.670)	0.3585	0.5086	Up
dihomo−gamma−linolenic acid	−0.106 (0.158)	0.045 (0.554)	0.3623	0.5121	Up
PC 38:6	−0.115 (0.263)	0.049 (0.401)	0.3718	0.5235	Up
Methylglutaryl−carnitine/Adipoyl−carnitine	0.156 (0.310)	−0.067 (0.560)	0.3763	0.5279	Down
PC 35:1	0.121 (0.521)	−0.052 (0.328)	0.3782	0.5287	Down
TG 52:4	−0.150 (0.456)	0.064 (0.515)	0.3913	0.5431	Up
alpha−Tocopherol	0.163 (0.601)	−0.070 (0.222)	0.3946	0.5431	Down
Dehydroepiandrosterone DHEA/ androstenolone	−0.247 (0.890)	0.106 (0.802)	0.3946	0.5431	Up

5−HETE	−0.117 (0.300)	0.050 (0.425)	0.3970	0.5431	Up
PC 34:0	−0.401 (0.838)	0.172 (0.803)	0.3970 (W)	0.5431	Up
PC 36:1	0.126 (0.284)	−0.054 (0.383)	0.3970 (W)	0.5431	Down
Aspartic acid	0.111 (0.356)	−0.048 (0.390)	0.4055	0.5527	Down
PC 36:3	0.174 (0.390)	−0.075 (0.675)	0.4137	0.5619	Down
LPE 20:5−sn2	0.204 (0.456)	−0.087 (0.800)	0.4192	0.5673	Down
PC 36:4	−0.065 (0.079)	0.028 (0.409)	0.4278	0.5769	Up
Glycochenodeoxycholic acid	0.183 (0.831)	−0.078 (0.595)	0.4346	0.5840	Down
PC 34:2 e	0.100 (0.391)	−0.043 (0.285)	0.4442 (W)	0.5930	Down
Progesterone	−0.172 (0.607)	0.074 (0.660)	0.4444	0.5930	Up
9−HODE/13−HODE	−0.118 (0.294)	0.051 (0.504)	0.4571	0.6075	Up
ChoE 22:4	0.129 (0.373)	−0.055 (0.537)	0.4584	0.6075	Down
Arachidonic acid	−0.101 (0.382)	0.043 (0.400)	0.4634	0.6120	Up
15−HETrE	0.123 (0.704)	−0.053 (0.363)	0.4659	0.6132	Down
PC 32:0	−0.090 (0.323)	0.039 (0.369)	0.4699	0.6145	Up
TG 52:3	−0.118 (0.371)	0.050 (0.499)	0.4704	0.6145	Up
LPE 16:1−sn2	0.161 (0.569)	−0.069 (0.666)	0.4717	0.6145	Down
SM 38:2	0.084 (0.284)	−0.036 (0.359)	0.4773	0.6177	Down
LPC 18:1−sn1	−0.083 (0.411)	0.035 (0.299)	0.4774	0.6177	Up
ChoE 17:0	0.190 (0.779)	−0.082 (0.773)	0.4810	0.6203	Down
LPE 20:5−sn1	0.181 (0.553)	−0.078 (0.810)	0.4869	0.6258	Down
Deoxycholic acid	−0.021 (1.058)	0.009 (0.594)	0.4940 (W)	0.6323	Up
Tetradecenoyl−carnitine	−0.135 (0.355)	0.058 (0.630)	0.4952	0.6323	Up
TG 54:2	0.112 (0.342)	−0.048 (0.515)	0.4975	0.6330	Down
PC 33:2	0.073 (0.351)	−0.031 (0.296)	0.5042	0.6394	Down
DG 36:1	−0.101 (0.400)	0.043 (0.481)	0.5264	0.6648	Up
N−Linoleoyl Ethanolamide	0.076 (0.385)	−0.032 (0.327)	0.5276	0.6648	Down
Threonic acid	0.098 (0.392)	−0.042 (0.471)	0.5315	0.6674	Down
PC 38:6 e	0.076 (0.311)	−0.033 (0.368)	0.5338	0.6681	Down
20−HETE	0.107 (0.364)	−0.046 (0.541)	0.5359	0.6685	Down
androsterone sulfate−iso4	−0.180 (0.718)	0.077 (0.422)	0.5466 (W)	0.6790	Up
LPC 15:0−sn1	0.071 (0.300)	−0.031 (0.355)	0.5478	0.6790	Down
PC 40:5	−0.086 (0.466)	0.037 (0.391)	0.5499	0.6794	Up
BAIBA	−0.123 (0.824)	0.053 (0.523)	0.5688	0.6999	Up
15−HETE	−0.097 (0.420)	0.042 (0.516)	0.5701	0.6999	Up
SM 40:0	−0.078 (0.542)	0.034 (0.332)	0.5746	0.7019	Up
ChoE 20:5	0.116 (0.461)	−0.050 (0.638)	0.5754	0.7019	Down
PC 36:2	0.070 (0.309)	−0.030 (0.383)	0.5795	0.7045	Down
Dodecenoyl−carnitine	0.092 (0.306)	−0.039 (0.536)	0.5855	0.7096	Down
LPC 18:0−sn1	−0.060 (0.203)	0.026 (0.361)	0.5924	0.7158	Up
ChoE 22:5	0.072 (0.423)	−0.031 (0.374)	0.5946	0.7162	Down
N−stearoyl−ethanolamine	−0.075 (0.241)	0.032 (0.456)	0.5984	0.7185	Up
Taurine	0.100 (0.068)	−0.043 (0.442)	0.6015 (W)	0.7200	Down
PC 34:3 e	−0.057 (0.371)	0.024 (0.293)	0.6050	0.7218	Up
LPE 22:5−sn1	0.116 (0.749)	−0.050 (0.617)	0.6107	0.7264	Down
PC 32:2	0.078 (0.354)	−0.033 (0.477)	0.6146	0.7288	Down
SM 42:2	0.063 (0.391)	−0.027 (0.347)	0.6166	0.7289	Down
Chenodeoxycholic acid	−0.140 (1.073)	0.060 (0.678)	0.6188	0.7293	Up
TG 46:2	−0.092 (0.378)	0.039 (0.595)	0.6268	0.7364	Up

5−Aminolevulinic acid	0.079 (0.539)	−0.034 (0.456)	0.6345	0.7424	Down
Fumaric acid	−0.046 (0.153)	0.020 (0.456)	0.6358	0.7424	Up
PC 38:4	−0.045 (0.153)	0.019 (0.448)	0.6384	0.7432	Up
Glycocholic acid	0.118 (0.760)	−0.051 (0.717)	0.6413	0.7443	Down
lauric acid	−0.074 (0.278)	0.032 (0.518)	0.6447	0.7457	Up
Arachidic acid	−0.101 (0.798)	0.043 (0.559)	0.6464	0.7457	Up
SM 38:1	−0.068 (0.464)	0.029 (0.434)	0.6569	0.7555	Up
PC 32:1 e	−0.023 (0.383)	0.010 (0.357)	0.6590 (W)	0.7557	Up
Tauroursodeoxycholic acid	0.134 (0.977)	−0.058 (0.461)	0.6619	0.7566	Down
dihomo−gamma−linolenic acid−iso1	0.058 (0.382)	−0.025 (0.390)	0.6644	0.7566	Down
PC 37:6	0.070 (0.522)	−0.030 (0.443)	0.6658	0.7566	Down
TG 52:1	−0.076 (0.466)	0.033 (0.564)	0.6828	0.7738	Up
Cholic acid	0.107 (1.013)	−0.046 (0.728)	0.7064	0.7981	Down
3−Methylhistidine	−0.063 (0.518)	0.027 (0.488)	0.7152	0.8034	Up
PC 37:4	0.055 (0.332)	−0.023 (0.464)	0.7153	0.8034	Down
ChoE 22:6	0.050 (0.377)	−0.021 (0.435)	0.7321	0.8185	Down
androsterone sulfate−iso3	0.078 (0.608)	−0.033 (0.676)	0.7337	0.8185	Down
ChoE 18:3	0.051 (0.412)	−0.022 (0.446)	0.7352	0.8185	Down
12,13−DiHOME9	0.051 (0.316)	−0.022 (0.518)	0.7542	0.8372	Down
Dodecanoyl−carnitine	0.054 (0.388)	−0.023 (0.549)	0.7579	0.8390	Down
Taurolithocholic acid	−0.076 (0.940)	0.032 (0.645)	0.7674	0.8468	Up
PC 40:4	0.048 (0.242)	−0.021 (0.541)	0.7699	0.8468	Down
SM 36:1	−0.033 (0.332)	0.014 (0.321)	0.7716	0.8468	Up
PC 36:5 e	−0.080 (0.224)	0.034 (0.343)	0.7791 (W)	0.8514	Up
PC 38:3	0.044 (0.293)	−0.019 (0.508)	0.7803	0.8514	Down
PC 34:2	0.027 (0.259)	−0.012 (0.356)	0.8145	0.8863	Down
Glycodeoxycholic acid	0.063 (1.137)	−0.027 (0.594)	0.8173	0.8868	Down
SM 35:0	0.026 (0.454)	−0.011 (0.296)	0.8258	0.8924	Down
OxoODE−iso2	0.041 (0.392)	−0.018 (0.593)	0.8279	0.8924	Down
Octadecanoyl−carnitine	−0.035 (0.297)	0.015 (0.538)	0.8330	0.8924	Up
PC 30:0	−0.031 (0.479)	0.013 (0.406)	0.8356	0.8924	Up
Serotonin	0.064 (0.333)	−0.027 (0.550)	0.8411 (W)	0.8924	Down
ChoE 18:0	−0.246 (0.191)	0.106 (0.837)	0.8411 (W)	0.8924	Up
PC 35:2	0.105 (0.441)	−0.045 (0.420)	0.8411 (W)	0.8924	Down
Decenoyl−carnitine	−0.006 (0.423)	0.003 (0.481)	0.8411 (W)	0.8924	Up
dihomo−gamma−linolenic acid−iso2	0.047 (0.454)	−0.020 (0.773)	0.8473	0.8965	Down
PC 31:0	0.026 (0.516)	−0.011 (0.343)	0.8501	0.8969	Down
PC 33:1	−0.024 (0.464)	0.010 (0.362)	0.8587	0.9035	Up
ChoE 16:1	0.029 (0.603)	−0.012 (0.482)	0.8716	0.9146	Down
LPC 20:4−sn1	0.023 (0.270)	−0.010 (0.560)	0.8932	0.9312	Down
PC 38:5	0.015 (0.224)	−0.007 (0.367)	0.8945	0.9312	Down
SM 36:2	−0.017 (0.293)	0.007 (0.406)	0.8960	0.9312	Up
EpOME−iso3	−0.020 (0.447)	0.009 (0.484)	0.9020	0.9312	Up
Lithocholic acid	0.122 (0.280)	−0.052 (0.792)	0.9044 (W)	0.9312	Down
Glyceric acid	0.061 (0.567)	−0.026 (0.418)	0.9044 (W)	0.9312	Down
PC 34:1	−0.015 (0.307)	0.006 (0.377)	0.9068	0.9312	Up
DiHODE−iso1	0.023 (0.455)	−0.010 (0.619)	0.9068	0.9312	Down

EpOME−iso2	0.015 (0.429)	−0.006 (0.433)	0.9194	0.9377	Down
TG 54:3	0.017 (0.349)	−0.007 (0.531)	0.9204	0.9377	Down
3−Hydroxybutiric acid	−0.020 (0.420)	0.008 (0.621)	0.9205	0.9377	Up
TG 54:4	0.015 (0.461)	−0.007 (0.484)	0.9255	0.9403	Down
HpEPE	0.014 (0.298)	−0.006 (0.514)	0.9287	0.9410	Down
LPC 20:2−sn1	−0.007 (0.212)	0.003 (0.345)	0.9519	0.9619	Up
PC 34:4	−0.007 (0.332)	0.003 (0.452)	0.9634	0.9710	Up
9,10−DiHOME12	−0.036 (0.464)	0.016 (0.603)	0.9671 (W)	0.9722	Up
LPE 22:6−sn2	0.005 (0.492)	−0.002 (0.556)	0.9798	0.9818	Down
PC 33:0	0.003 (0.467)	−0.001 (0.431)	0.9817	0.9818	Down

DG, diglycerides; ChoE, cholesteryl ester; HDHA, hydroxydocosahexaenoic acid; LPE, lysophosphatidylethanolamine; LPI, lysophosphatidylinositol; LPC, lysophosphatidylcholines; PC, phosphatidylcholines; SM, sphingomyelines; TG, triglycerides.
